# Exposure Characteristics of the Analogous β-Carboline Alkaloids Harmaline and Harmine Based on the Efflux Transporter of Multidrug Resistance Protein 2

**DOI:** 10.3389/fphar.2017.00541

**Published:** 2017-08-21

**Authors:** Shuping Li, Yunpeng Zhang, Gang Deng, Yuwen Wang, Shenglan Qi, Xuemei Cheng, Yueming Ma, Yan Xie, Changhong Wang

**Affiliations:** ^1^The MOE Key Laboratory for Standardization of Chinese Medicines, The SATCM Key Laboratory for New Resources and Quality Evaluation of Chinese, Institute of Chinese Materia Medica, Shanghai University of Traditional Chinese Medicine Shanghai, China; ^2^Shanghai R&D Centre for Standardization of Chinese Medicines Shanghai, China; ^3^Laboratory of Pharmacokinetics, Shanghai University of Traditional Chinese Medicine Shanghai, China; ^4^Research Center for Health and Nutrition, Shanghai University of Traditional Chinese Medicine Shanghai, China

**Keywords:** β-carboline alkaloid, transport, metabolism, pharmacokinetic, MRP2

## Abstract

Harmaline and harmine occur naturally in plants and are distributed endogenously in human and animal tissues. The two β-carboline alkaloids possess potential for treating Alzheimer's disease, Parkinson's disease, depression and other central nervous system diseases. However, studies have showed that the two compounds have similar structures but with quite different bioavailability. The aim of this study was to elucidate the exposure difference and characterize the *in vitro* transport, metabolism, and pharmacokinetic properties of harmaline and harmine. The results showed that the harmaline and harmine transport across the Caco-2 and MDCK cell monolayers was varied as the time, concentration, pH and temperature changed. The absorption of harmaline and harmine was significantly decreased when ES (OATPs inhibitor), TEA (OCTs/OCTNs substrate), NaN_3_ (adenosine triphosphate inhibitor), or sodium vanadate (ATPase Na^+^/K^+^-dependent inhibitor) was added. However, when given MK571 and probenecid (the typical MRP2 inhibitor), the *P*_*appAB*_ of harmine was increased (1.62- and 1.27-folds), and the efflux ratio was decreased from 1.59 to 0.98 and from 1.59 to 1.19, respectively. In addition, the uptake ratio of harmine at 1 μM was >2.65 in the membrane vesicles expressing human MRP2. Furthermore, harmine could slightly up-regulate the expression of MRP2, which implying harmine might be the substrate of MRP2. Particularly, the *CL*_*int*_-value for harmine was ~1.49-folds greater than that of harmaline in human liver microsomes. It was worth noting that the *F*-value of harmine was increased 1.96-folds after harmine co-administration with probenecid. To summarize, comprehensive analysis indicated that harmaline and harmine were absorbed by transcellular passive diffusion and a pH- and Na^+^-dependent mechanism might be mediated by OATPs and OCTs/OCTNs. MRP2 but MDR1 or BCRP might be involved in the transport of harmine. Furthermore, harmine was more unstable and easily metabolized than harmaline. All these findings suggested that harmine not only appears be an MRP2 substrate, but also possesses weak metabolic stability, and eventually leads to a low oral bioavailability. Taken together, the elucidated absorption, transport, metabolism as well as pharmacokinetic characteristics of harmaline and harmine provide useful information for designing delivery systems, pharmacological applications and avoiding drug-drug interactions.

## Introduction

The analogous β-carboline alkaloids, harmaline, and harmine (Figure S1), dominant pharmacological ingredients of plants *Peganum harmala* L., *Passiflora incarnata* L., and *Banisteriopsis caapi* (Spruce ex Griseb.). Morton, are ubiquitously available in a variety of medicinal plants (Khan et al., 2013; Ingale and Kasture, 2014; Stanković et al., 2015; Li et al., 2017). Harmaline and harmine are endogenously produced in human and animal tissues as a low molecular weight product of secondary metabolism (Li et al., 2016). They could affect the content of neurotransmitters by strong inhibition of monoamine oxidase (Jiang et al., 2015), acetylcholinesterase (Liu et al., 2014), and myeloperoxidase (Bensalem et al., 2014). Furthermore, they possess capability to bind to imidazoline, serotonin, dopamine, opiate, and benzodiazepine receptors, which cause physiological, biochemical, and behavioral changes in human and animals (Wu et al., 2009; Zhao et al., 2011, 2012). They have attracted much attention in relation to their biological activities, and their proposed use for treatment of neurological disorders on the basis of activity as inhibitors of acetylcholinesterase *in vitro* (Khan et al., 2013; Zhao et al., 2013; Li et al., 2017). Since the biological efficacy depends significantly on the oral bioavailability of drugs, it is important to understand the molecular properties such as metabolic stability and cell permeability that limit the oral bioavailability.

In view of the pharmacokinetic study of harmaline and harmine, it was interesting to find that significant difference presented in the absolute bioavailability (*F*) of the two analogs among different experimental animals, such as rats, dogs and mice. The *F*-values were 63.22 and 24.99% for harmaline, while only 4.96% and 5.33% for harmine after oral administration in rats and dogs with the total alkaloid extracts (140 mg/kg) from *P. harmala* (Zhang, 2013; Shi et al., 2014). According to another study of Guan et al. (2001), the *F*-value of harmine was 3% in rats by gastric *gavage* (20 mg/kg). Following an intraperitoneally injection, the *F*-values of harmaline were 73.7, 98.2, 68.6, 60.0% in wild-type and CYP2D6-humanized mice with either 5 or 15 mg/kg (Wu et al., 2009). Generally speaking, the oral bioavailability of harmine was much lower than that of harmaline in different animals. Furthermore, Khan et al. (2004) reported that the intestinal transport of β-carboline alkaloids (harmaline, harmine, harmalol, harmol, and harmane) in the concentration range of 250–500 μM using a human colon carcinoma (Caco-2) cell monolayers. It revealed that the efflux ratios (*ER*) of harmaline and harmine were <1.0, suggesting a passive diffusion mechanism for their transport across the Caco-2 monolayer. However, composite evidences indicate that these alkaloids possess a strong cytotoxicity to various cell lines, particularly harmine (Li et al., 2016). The drug concentration (250–500 μM) may exceed the safe dosage in the Caco-2 cells, which will result in damage to cells and then change the activity of transporters or the permeability of the cell monolayers. Additionally, it was far from the illustration of their differences in the oral bioavailability based on the above results. Hence, a systematic and rigorous study on the transport mechanism of these alkaloids should be conducted to clarify the exposure characteristics.

Cell-based assays for evaluation of permeability can glean insight into potential issues with intestinal transport. Several cell culture models for determination of the *in vitro* intestinal permeability have been developed and currently gained great popularity. Amongst various models, Caco-2 and Madin-Darby canine kidney (MDCK) cells have been extensively used as the *in vitro* model for evaluating drug intestinal transport mechanism (Volpe, 2011; Chen et al., 2014; Shen et al., 2015). In the late 1990s, transfected MDCK cell lines have been reported to express high levels of multidrug resistance gene 1 (MDR1), breast cancer resistance protein (BCRP) and multidrug resistance-associated protein isoform 2 (MRP2) on the apical side of the polarized cell monolayer. Furthermore, inverted membrane vesicles with over-expressed transporters allow a way to study the transport mechanism of drugs. The vesicle-based assay possesses a higher throughput and convenience compared to whole cell assays since they can be prepared in large batches and cryopreserved for later use. The interaction data generated in the vesicle system can be used to predict the *in vivo* transporter-mediated disposition and possible drug-drug interactions (Deng et al., 2016). Therefore, the combined use of the Caco-2, MDCK and transfected MDCK cells, as well as specific inverted membrane vesicles, could yield clear advantages in the transport study of these β-carboline alkaloids.

The purpose of this study was to comprehensively clarify the exposure difference and characterize the *in vitro* transport, metabolism, and pharmacokinetic properties of the analogous harmaline and harmine. In the present study, the bidirectional transport of harmaline and harmine across the Caco-2, MDCK, MDCK-MRP2 cells in the absence and presence of influx and efflux transporter inhibitors or substrates was investigated. Considering the structural features of the two alkaloids and the transporter expression of the Caco-2 and MDCK cells, the experiments were tentatively conducted on the SLC influx (OATs, OATPs, OCTs, OCTNs, MCTs, SGLT1, PEPT1, etc.) and ABC efflux transporters (MDR1, BCRP, MRP2; van Montfoort et al., 2003; Koepsell and Endou, 2004; Seithel et al., 2006; Koepsell et al., 2007; Volpe, 2011; Koepsell, 2013; Chen et al., 2014; Arimany-Nardi et al., 2015; Shen et al., 2015). Remarkably, many members are present in the groups of OATs (OAT1, OAT2, OAT3, OAT4, and OAT5), OATPs (OATP-A, OATP-B, OATP-C, OATP-D, OATP-E, etc.), OCTs (OCT1, OCT2, and OCT3), OCTNs (OCTN1, OCTN2, and OCTN3), and MCTs (MCT1, MCT2, MCT3, MCT4, MCT5, etc.; van Montfoort et al., 2003; Koepsell and Endou, 2004; Shen et al., 2015). Evaluations were also carried out using inverted membrane vesicles to determine the effect of efflux transporters on the alkaloids transport. Moreover, *in vitro* metabolic stability and *in vivo* pharmacokinetic properties of the two analogs were underway to characterize. The results of the current study will be helpful in evaluating the pharmacology and toxicology of the β-carboline alkaloids in further development, and could provide significant information for clinical practice.

## Materials and methods

### Materials

Harmaline, harmine, tacrine hydrochloride (internal standard, IS), verapamil hydrochloride, quinidine, Ko143, apigenin, MK571, probenecid, sodium vanadate, cimetidine, estrone-3-sulfate (ES), tetraethylamine (TEA), phloretin, sodium azide (NaN_3_), phloridzin, glycylsarcosine (Gly-Sar), dimethyl sulfoxide (DMSO), ethylenediaminetetraacetic acid (EDTA), and all other chemicals used were purchased from Sigma Aldrich Co., Ltd (St. Louis, MO, USA). Dulbecco's modified eagle's medium (DMEM), phosphate buffered saline (PBS), Hanks' balanced salt solution (HBSS) with calcium chloride and magnesium chloride, HBSS without calcium chloride and magnesium chloride, heat inactivated fetal bovine serum (FBS), penicillin-streptomycin solution, non-essential amino acids (NEAA), 0.25% trypsin-EDTA, and other reagents were purchased from Gibco Lab (Grand Island, NY, USA). The cell counting kit-8 (CCK-8), BCA protein quantification kit, PBST, RIPA lysis buffer, bovine serum albumin (BSA), 30% acrylic amide, 10% SDS, TEMED, and sample loading buffer (4X) were purchased from YEASEN Biotechnology Co., Ltd (Shanghai, China). Protease inhibitor and phospholipase inhibitor were purchased from Roche Applied Science (Foster City, CA, USA). PVDF membrane, Millicell® ERS-2, 24-well cell culture standing inserts, and Immobilon™ Western chemiluminescent HRP substrate were purchased from Millipore (Billerica, MA, USA). Marker, rabbit anti-MRP2 and anti-glyceraldehyde 3-phosphate dehydrogenase (GAPDH), and HRP-conjugated anti-rabbit IgG antibodies were purchased from Abcam Technology (Cambridge, MA, USA). Acetonitrile, methanol, and formic acid of HPLC grade were purchased from Fisher Scientific Co. (Santa Clara, CA, USA). Deionized water was purified using a Milli-Q Academic System (Millipore Corp., Billerica, MA, USA). All other chemicals were of analytical grade.

### Animals

Fifty-six pathogen-free Sprague-Dawley adult rats comprising 28 males and 28 females (weighing within the range of 200–250 g) were obtained from Drug Safety Evaluation and Research Center of Shanghai University of Traditional Chinese Medicine. Animals were raised under an environmentally controlled breeding room for 7 days before commencing the experiments. Animals were housed in a well-lighted air-conditioned room (25 ± 1°C) under standard environmental conditions (12 h light-dark cycles) and given free access to rodent chow and tap water prior to the study. Rats were fasted for 12 h and provided free access to water prior to the experiments. All animal-use procedures were in accordance with the regulations for animal experimentation issued by the State Committee of Science and Technology of the People's Republic of China on 14 November 1988 and approved by the Animal Ethics Committee of Shanghai University of Traditional Chinese Medicine (No. SUTCM-2011-1107; Approval date: 10 November, 2011).

### Cell culture

Caco-2, MDCK and MDCK-MRP2 cells were donated by Prof. Yan Xie and Yueming Ma from Shanghai University of Traditional Chinese Medicine. The cells were grown in culture dishes (Corning® Costar, Cambridge, MA, USA) using DMEM supplemented with 10% FBS, 1% NEAA and 1% penicillin-streptomycin solution. The cells were cultured in 5% CO_2_ and 95% O_2_ with 90% relative humidity at 37°C. The medium was replaced every other day during incubation. The cells were passaged every 3–4 days between 70 and 80% confluence at a 1:5 split ratio using 0.25% trypsin-EDTA. For the transport experiments, the cells from passages between 30 and 45 were seeded at 1 × 10^5^ cells/cm^2^ onto permeable polycarbonate inserts (0.45 μm pore size, seeding surface of 0.6 cm^2^, Millipore, MA, USA) in 24-well plastic plates. The media in the culture plates were changed every other day for the first week after seeding and were replaced daily afterward. The integrity of the cell monolayers and tight junctions (TJ) were tested and confirmed by measuring transepithelial electrical resistance (TEER) with a Millicell® ERS-2 electrode. The cells were used for the transport experiments 21–28 (Caco-2) or 5–7 (MDCK and MDCK-MRP2) days after seeding, and only monolayers with a TEER-value above 420 Ω cm^2^ were used during the studies.

### Cytotoxicity assay

The cytotoxicity of harmaline and harmine was measured by CCK-8 assay. In brief, the cells were seeded on 96-well plates at 1 × 10^5^ cells/mL and cultured in 100 μL of culture medium at incubator for 24 h. The medium in each well was then replaced with 100 μL of medium containing harmaline or harmine at the following concentrations: 0.5, 1, 2.5, 5, 25, and 100 μM. After 12 h incubation, 10 μL of CCK-8 dye was added to each well, and cells were incubated for another 2 h. Cell viability (%) was calculated using the following Equation (1):

(1) $$\begin{array}{l} \text{Cell viability }(\%)=({\text{OD}}_{\text{sample}}-{\text{OD}}_{\text{blank}})\text{/}\\ \;({\text{OD}}_{\text{control}}-{\text{OD}}_{\text{blank}})\;\times 100\% \end{array}$$

OD_sample_ refers to the absorbance of a well with treated cells and CCK-8. OD_blank_ refers to the absorbance of a well with medium and CCK-8 but without cells. OD_control_ refers to the absorbance of a well with untreated cells and CCK-8. The absorbance at 450 nm was measured by a microplate reader (Biotek Instrument; Gene Co., Ltd., VT, USA), and the results are presented as mean ± *SD* from triplicate wells.

### Transport study

The apical (AP) and basolateral (BL) sides are typical of polarized Caco-2, MDCK and MDCK-MRP2 epithelial cells. Before the experiments, the polarized cell monolayers were washed twice with warm HBSS solution and subsequently preincubated (37°C, 30 min). Afterward, HBSS solution on both sides of the cell monolayers was removed using a mini desktop vacuum pump. To measure the AP to BL permeability (absorptive transport), 400 μL HBSS containing harmaline or harmine was added to the AP side, while 600 μL blank HBSS was added to the BL side. To measure the BL to AP permeability (secretive transport), 600 μL HBSS containing harmaline or harmine was added to the BL side and 400 μL blank HBSS was added to the AP side. The harmaline or harmine solution was freshly prepared in DMSO. The final DMSO concentration in the HBSS or drug mixture was below 0.5%. The monolayers were incubated at 37°C, and 100 μL samples were taken at 30, 60, 90, and 120 min from the acceptor compartment and immediately replaced with the same volume of prewarmed fresh blank HBSS. TEER measurements for assessing the membrane integrity took place before and after the experiments.

Harmaline and harmine transport was assessed in both directions at different concentrations (1, 2, and 5 μM). The effect of pH on harmaline or harmine transport in both directions was studied using the following pH in the acceptor or donor compartment: 5.5/5.5 and 7.4/7.4. The harmaline or harmine transport at 4 and 37°C in both directions was evaluated to investigate the temperature effect. To reveal the paracellular harmaline or harmine transport, the cell monolayer was preincubated with 5 mM EDTA in HBSS without Ca^2+^ and Mg^2+^ for 30 min. Subsequently, the harmaline or harmine with 5 mM EDTA in HBSS without Ca^2+^ and Mg^2+^ was added to the AP or BL compartment.

The transport specificity experiments were performed by testing inhibition of transport by selective inhibitors or substrates of the chosen transporters. To investigate the roles of influx and efflux transporters in harmaline or harmine transport, 50 μM sodium vanadate (Na^+^/K^+^-ATPase), 10 mM NaN_3_ (adenosine triphosphate, ATP), 50 μM cimetidine (OATs), 50 μM ES (OATPs), 5 mM TEA (OCTs/OCTNs), 0.3 mM phloretin (MCTs), 0.5 mM phloridzin (SGLT1), 10 mM Gly-Sar (PEPT1), 100 μM verapamil and quinidine (MDR1), 50 μM MK571 and 200 μM probenecid (MRP2), 10 μM Ko143, and 25 μM apigenin (BCRP) were added at both AP and BL sides of monolayers. After 30 min preincubation, these compounds with harmaline or harmine (2 μM) were added to either the AP or BL side and blank HBSS buffer was added to the other side, respectively. As a control, in each inhibition experiment harmaline or harmine transport was also assessed in the absence of any inhibitor or substrate.

The apparent permeability coefficients (*P*_*app*_) and *ER* were calculated as Equations (2) and (3):

(2) $$P_{app}=(dQ/dt)/(A\times C_{0})$$

(3) $$ER=P_{appBA}/P_{appAB}$$

where *dQ/dt* is the steady-state flux, *A* is the membrane surface area, and *C*_0_ is the initial drug concentration in the donor compartment. *P*_*appBA*_ is the *P*_*app*_-value measured in BL to AP direction and *P*_*appAB*_ is the *P*_*app*_-value measured in AP to BL direction. It indicated the involvement of an active transport process when the *ER-*value is above 1.5 (Hubatsch et al., 2007).

### Vesicular transport

The vesicular transport assay was implemented as described in the GenoMembrane™ vesicular transport kit protocol (GenoMembrane, Kanagawa, Japan) with minor changes. Briefly, vesicle stocks were thawed and diluted with assay buffer (50 mM MOPS-Tris, 70 mM KCl, and 7.5 mM MgCl_2_). Ten microliters harmaline or harmine at the concentration of 1 or 10 μM was mixed with 5 μL assay buffer and 20 μL 10 mM MgATP solution; 10 μL vesicle was mixed with 5 μL assay buffer; after 5 min preincubation, the two mixtures were then mixed and incubated for another 5 min. MgAMP instead of MgATP was used as a control. Apart from these, 4 mM glutathione was also included in BCRP and MRP2 assays. Transport was terminated with ice cold assay buffer, and then samples were quickly transferred to a filter plate. Wells were immediately washed 5 times with 200 μL assay buffer and dried. The vesicles on the plate were dissolved in 50 μL of 80% methanol and centrifuged at 2,000 rpm for 2 min after collection, and repeating the above procedures. Mixed the two filtrates together and added the pre-cooled methanol. After centrifugation at 12,000 rpm for 5 min at 4°C, a 5 μL aliquot of supernatant was injected into the UPLC-ESI-MS/MS system for analysis. Furthermore, the uptake of NMQ (MDR1), LY (BCRP), and E_2_17βG (MRP2) was used to evaluate the activity of specific vesicles.

### Western blot analysis

The Caco-2 cells were seeded into 6-well plates at a density of 1 × 10^5^ cells/mL and cultured for 24 h. After treatment with harmaline or harmine for 48 h with their concentrations of 1, 2, and 5 μM, cells were washed 3 times with ice-cold PBS and lysed on ice with RIPA lysis buffer containing protease inhibitor and phosphatase inhibitor. Protein lysate was collected by centrifugation at 12,000 rpm at 4°C for 10 min, and the total protein content was determined using BCA protein assay kit. Proteins were then mixed with a quarter volume of loading buffer and heated at 100°C for 5 min. Approximately 20 μg of the total protein content was separated by SDS-PAGE through an 8% acrylamide gel and transferred to PVDF membranes. After washing with PBST, the membrane was blocked with 5% fat-free milk in PBST for 1 h at room temperature (RT) and then incubated with anti-MRP2 (1:500) and anti-GAPDH (1:5,000) overnight at 4°C. Afterwards, the membranes were rinsed with PBST and incubated with HRP-conjugated anti-rabbit (1:5,000) secondary antibody for 2 h at RT. After complete washing with PBST, the protein bands were visualized with ECL prime kit (GE Healthcare, NA, UK).

### Drug analysis

#### Extraction procedure

A convenient and rapid precipitation method was used to prepare the transport and following plasma samples. A 100 μL aliquot of sample was added with 400 μL acetonitrile containing IS in a 1.5 mL centrifuge tube and then vortex-mixed for 1.0 min; subsequently, the mixture was centrifuged at 12,000 rpm for 10 min at 4°C. Up to 400 μL of supernatant was transferred to another clean tube and evaporated to dry at 37°C under a slight stream of nitrogen. The dried residue was reconstituted with 80 μL of 9% acetonitrile and vortexed for 1.0 min. After centrifugation at 12,000 rpm for 10 min at 4°C, a 5 μL aliquot of supernatant was injected into the UPLC-ESI-MS/MS system for analysis.

#### UPLC-ESI-MS/MS analysis

Harmaline and harmine concentrations were simultaneous quantitative determined on a Waters-ACQUITY™ UPLC system (Waters Corp., Milford, MA, USA) using an ACQUITY UPLC BEH C_18_ column (50 × 2.1 mm, 1.7 μm particle size). Mass spectrometric detection was performed using a triple quadrupole mass spectrometer (Waters Corp., Milford, MA, USA) equipped with electrospray ionization in positive ionization mode, and all other instrumental parameters were set according to our previous study (Li et al., 2016). The UPLC-ESI-MS/MS method was well-validated (data not shown) and the analytical method was successfully applied to determine the concentration of harmine and harmaline in the HBSS buffers. Figure S2 presents the representative of typical MRM chromatograms of blank HBSS, blank HBSS spiked with harmine, harmaline and IS, and IS-spiked HBSS sample collected at 30 min after administration of harmine and harmaline.

#### Metabolic stability of harmaline and harmine in human liver microsomes

Harmaline or harmine (2 μM) was incubated with human liver microsomes (0.5 mg protein/mL) for 0, 5, 10, 20, 30, 45, 60, 90 min using our previous method (Li et al., 2016). The residual percentage of harmaline or harmine was plotted vus incubation time. The depletion half-life (*T*_*1/2*_) of harmaline or harmine was calculated by regression analysis of semilogarithmic plots. Intrinsic clearance (*CL*_*int*_) of harmaline or harmine was estimated according to the equation (4) from the *in vitro T*_*1/2*_, incubation volume (*V*), and mass of microsomal proteins in the incubation mixture (*P*) (Liu et al., 2010).

(4) $$CL_{int}=(0.693/T_{1/2})\times (V/P)$$

#### Pharmacokinetics

The validated method was applied to the pharmacokinetic study of harmaline and harmine in rats after single intravenous and intragastric administration of harmaline, harmine, or harmine co-administration with probenecid. Forty rats with 20 males and 20 females were randomly divided into five groups of eight rats in each. Harmaline or harmine was administered to eight rats by *gavage* at a dose of 40.0 mg/kg, and eight rats were administered via the caudal vein at a dose of 3.3 mg/kg, which was dissolved in physiological saline with germicidal treatment; the eight other rats were orally administered with the mixture of harmine (40.0 mg/kg) and probenecid (20.0 mg/kg). Approximately 0.25 mL of blood sample was collected from the angular vein of each conscious rat and transferred into heparinized tubes at 0 (predose), 0.03, 0.08, 0.25, 0.5, 0.75, 1.0, 2.0, 4.0, 8.0, 12.0, and 24.0 h after administration. Rats had free access to water after 4.0 h of blood sample collection. Serial blood samples were immediately centrifuged at 3,000 rpm for 15 min, and 100 μL of the supernatant plasma layer was transferred into another new 1.5 mL centrifuge tube and stored at −80°C until analysis.

The plasma concentrations of harmaline and harmine were directly calculated by their calibration curves accordingly. The plasma concentration vs. time curves were plotted, and all the pharmacokinetic parameters of harmaline and harmine, such as absorption rate constant (*K*_*a*_), distribution rate constant (*K*_*d*_), elimination rate constant (*K*_*e*_), absorption half-life (*T*_*1/2a*_), distribution half-life (*T*_*1/2d*_), elimination half-life (*T*_*1/2e*_), apparent volume of distribution (*V*_*d*_), clearance rate (*CL*), area under the plasma concentration-time curve from zero to time *t* (*AUC*_0−*t*_), area under the plasma concentration-time curve from zero to infinity (*AUC*_0−∞_), and mean residence time (*MRT*), were processed using the non-compartmental pharmacokinetic data analysis software program of PK solution 2.0™ (Summit Research Services, USA). The maximum peak concentration (*C*_*max*_) and time of maximum plasma concentration (*T*_*max*_) were obtained directly from the observed concentration vs. time data. The *F*-value of harmaline or harmine was calculated by the ratios of dose-normalized *AUC*_0−∞_after oral and intravenous dosing as the following Equation (5):

(5) $$F=(AUC_{0-\infty ,oral}\times {\text{Dose}}_{iv})/(AUC_{0-\infty ,iv}\times {\text{Dose}}_{oral})\times 100\%$$

### Excretion in rats

Sixteen rats with eight males and eight females were put into metabolic cages individually and then fasted overnight till 2 h after oral administration of harmaline and harmine at a dose of 40.0 mg/kg. Access to water was maintained all the time. Samples including blank urine and feces before dosing, urine and feces from 0 to 24 h, 24 to 48 h, and 48 to 72 h after dosing were collected and stored at −80°C until analysis.

Urine samples were thawed and an aliquot of 100 μL was processed for detection as described in Section Drug Analysis. Feces were homogenized and then weighed, added 6-folds of acetonitrile, extracted by ultrasonic wave for 1 h and then centrifuged. An aliquot of 100 μL was also processed for detection as described in Section Drug Analysis.

### Data analysis

Data analysis was carried out with SPSS version 17.0 software, and the data were expressed as the mean ± *SD*. To compare the two groups, a two tailed unpaired Student's *t*-test was employed. *P* < 0.05, *P* < 0.01, and *P* < 0.001 were considered statistically significant.

## Results

### Cytotoxicity assay

Harmaline and harmine were tested with CCK-8 assay for possible cytotoxic effects in the Caco-2 and MDCK cell lines. Over 90% of the Caco-2 and MDCK cells were viable when up to 25 μM for harmaline or 5 μM for harmine was used during the experiments (Figure S3). According to these results, it was confirmed that none of harmaline and harmine at the tested concentrations (1, 2, and 5 μM) showed toxicity or decreased cell viability.

### Transport study

#### Transport characteristics

The transcellular harmaline and harmine transport across the Caco-2 and MDCK cell monolayers was investigated as time, concentration, pH, temperature changed, and the role of paracellular pathway.

#### Effect of time and concentration

**Figure 1:**
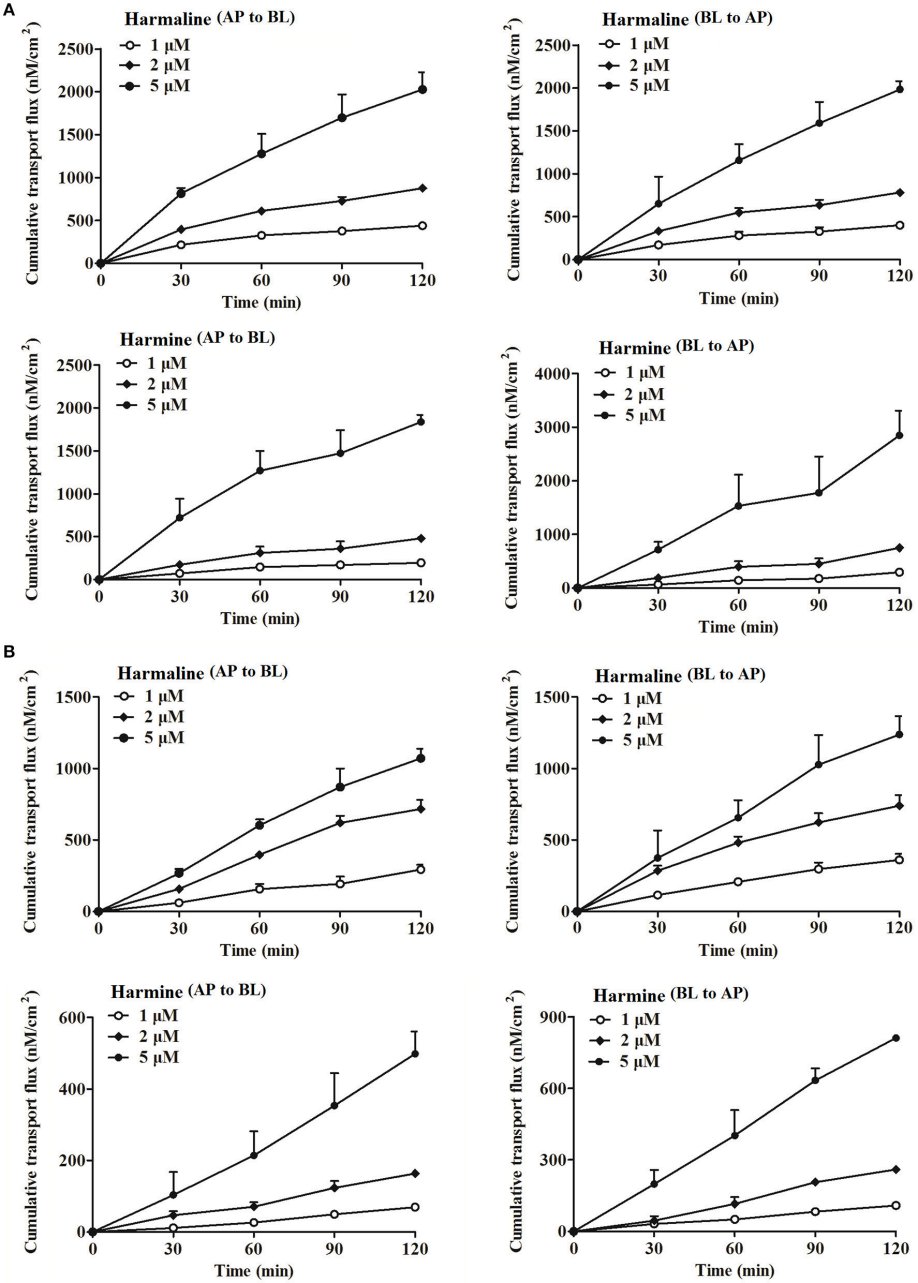
The cumulative transport fluxes of harmaline and harmine in the Caco-2 **(A)** and MDCK **(B)** cell monolayers in both directions (AP to BL and BL to AP) at different times and concentrations. Here, the AP to BL direction (absorptive transport): harmaline or harmine was added to the AP side and samples were collected from the BL side; the BL to AP direction (secretive transport): harmaline or harmine was added to the BL side and samples were collected from the AP side. Data represent the mean ± *SD* from three replicates.

A total of three concentrations (1, 2, and 5 μM) were used to examine harmaline and harmine transport across the Caco-2 and MDCK cell monolayers at 37°C with the incubation time from 0 to 120 min on either the AP or BL side. No obvious change in TEER-value was observed among the concentration groups of harmaline and harmine during the 2 h experimental exposure, suggesting the integrity of the cell monolayers. As shown in Figure 1, the transport of harmaline and harmine in both directions increased gradually with time and concentration. Interestingly, the cumulative transport fluxes of harmine among the concentration groups were much higher in the BL to AP direction than those in the AP to BL direction. The *P*_*app*_ and *ER*-values of harmaline and harmine in both directions are summarized in Table 1. As the concentration increased, the *P*_*app*_-values of harmaline and harmine increased in both directions. In the AP to BL direction, the harmaline and harmine permeabilities were all lower than that of propranolol, a transcellular flux marker with *P*_*appAB*_-value of (41.90 ± 3.30) × 10^−6^ cm/s (Shen et al., 2015). The *ER*-values of harmaline were <1.5 at the tested concentrations, and it can be confirmed that harmaline was transported mainly by passive diffusion. However, the BL to AP harmine transport was 1.5- to 1.6-folds higher than the transport in the opposite direction. The *ER*-values at different concentrations were all >1.5, suggesting that an active transport process was involved (Hubatsch et al., 2007). Harmine might be a substrate for efflux transporters, such as MDR1, BCRP, and MRP2, which can participate in the efflux process, resulting in lower concentrations of harmine in the receptor compartment.

**Table 1:** The bidirectional *P*_*app*_-values of harmaline and harmine across the Caco-2 and MDCK cell monolayers (mean ± *SD, n* = 3).

**Cell**	**Drug**	**Initial concentration (μM)**	***P_*appAB*_*(1 × 10^−6^ cm/s)**	***P_*appBA*_*(1 × 10^−6^ cm/s)**	***ER***
Caco-2	Harmaline	1	27.45 ± 1.44	25.57 ± 1.95	0.93
		2	27.72 ± 1.06	26.42 ± 0.49	0.95
		5	30.10 ± 3.01	30.98 ± 1.40	1.03
	Harmine	1	15.13 ± 0.73	22.84 ± 0.25	1.51
		2	18.78 ± 1.26	29.34 ± 1.21	1.56
		5	31.15 ± 1.42	48.29 ± 2.81	1.55
MDCK	Harmaline	1	15.81 ± 1.76	19.47 ± 2.25	1.23
		2	20.36 ± 1.82	21.03 ± 2.11	1.03
		5	21.21 ± 1.32	24.53 ± 2.54	1.16
	Harmine	1	3.70 ± 0.14	5.81 ± 0.19	1.57
		2	4.60 ± 0.25	7.32 ± 0.19	1.59
		5	7.11 ± 0.89	11.58 ± 0.15	1.63

#### Effect of pH

**Figure 2:**
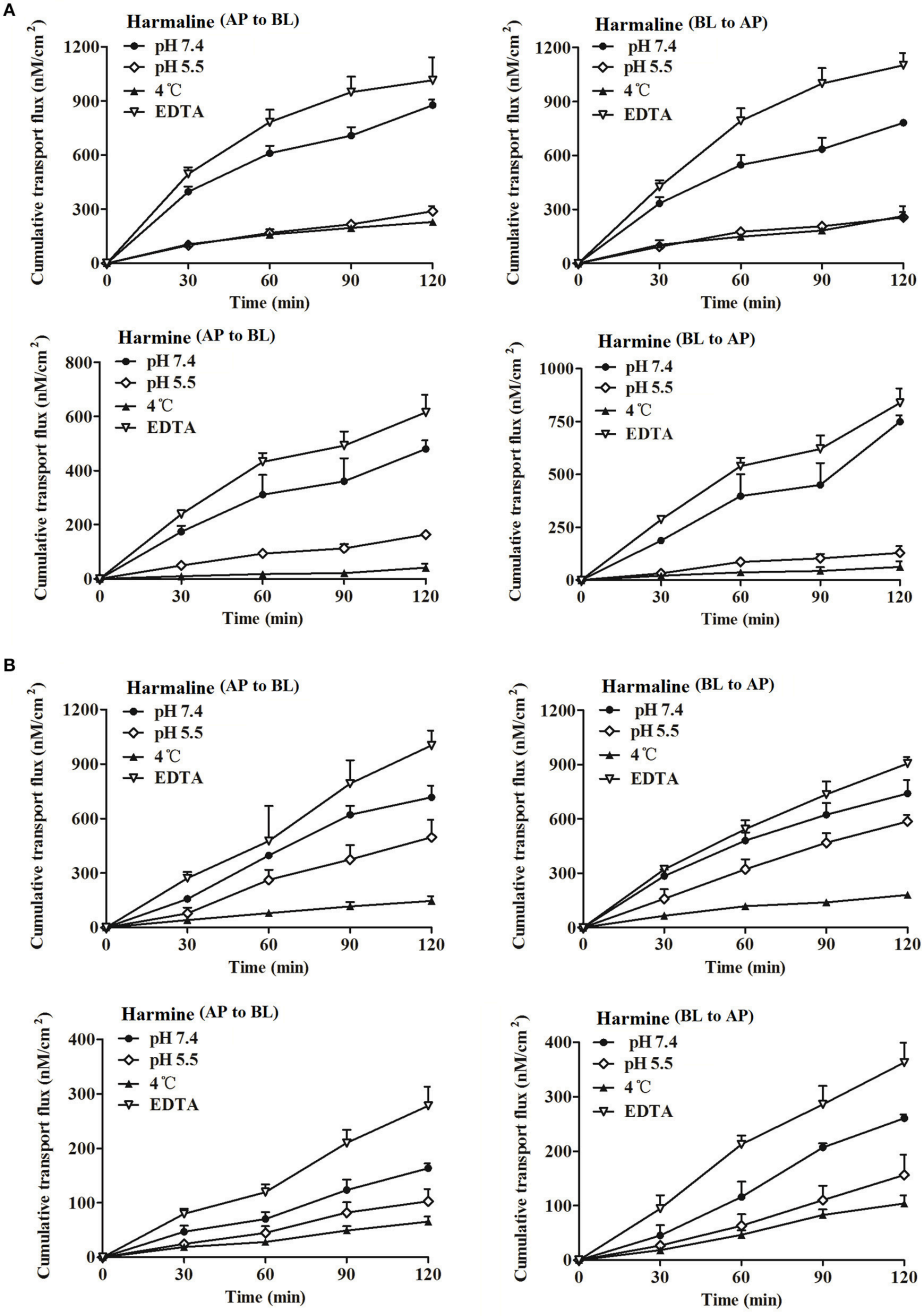
The cumulative transport fluxes of harmaline and harmine in the Caco-2 **(A)** and MDCK **(B)** cell monolayers in both directions (AP to BL and BL to AP) at different conditions (pH 7.4 or 5.5, 37 or 4°C, with or without EDTA). Data represent the mean ± *SD* from three replicates.

The effect of pH on harmaline and harmine (2 μM) bidirectional transport in the two cell monolayers were determined. As presented in Figure 2, the cumulative transport fluxes of harmaline and harmine at pH 5.5 were much lower than those at pH 7.4 in both directions. The *P*_*app*_-values of harmaline and harmine in bidirection are listed in Table 2. The *P*_*appAB*_-values of harmaline and harmine at pH 7.4 were significantly higher (3.65- and 3.56-folds) than those at pH 5.5 (*P* < 0.001) across the Caco-2 cell monolayers, and which were also obviously higher (1.56- and 2.05-folds) than those at pH 5.5 (*P* < 0.05) across the MDCK cell monolayers. Consequently, the data indicated that harmaline and harmine transport was pH-dependent, which might be related to the physicochemical properties of drugs.

**Table 2:** The bidirectional *P*_*app*_-values of harmaline and harmine in the Caco-2 and MDCK cell monolayers at different conditions (pH 7.4 or 5.5, 37 or 4°C, with or without EDTA; mean ± *SD, n* = 3).

**Cell**	**Drug**	**pH/Temperature**	***P_*appAB*_* (1 × 10^−6^ cm/s)**	***P_*appBA*_* (1 × 10^−6^ cm/s)**	***ER***
Caco-2	Harmaline	Control	27.72 ± 1.06	26.42 ± 0.49	0.95
		5.5	7.60 ± 0.74^***^	6.72 ± 0.76^***^	0.88
		4°C	7.59 ± 0.33^***^	8.71 ± 1.83^***^	1.15
		with EDTA	35.23 ± 2.68^*^	39.57 ± 2.10^*^	1.12
	Harmine	Control	18.78 ± 1.26	29.34 ± 1.21	1.56
		5.5	5.27 ± 0.29^***^	4.17 ± 1.03^***^	0.79
		4°C	1.58 ± 0.57^***^	2.41 ± 0.85^***^	1.52
		with EDTA	24.11 ± 2.53^*^	29.67 ± 2.22	1.23
MDCK	Harmaline	Control	20.36 ± 1.82	21.03 ± 2.11	1.03
		5.5	13.03 ± 2.54^*^	15.40 ± 0.91^*^	1.18
		4°C	5.40 ± 0.99^***^	6.65 ± 0.64^***^	1.23
		with EDTA	29.43 ± 1.45^*^	26.34 ± 0.93^*^	0.90
	Harmine	Control	4.60 ± 0.25	7.32 ± 0.19	1.59
		5.5	2.24 ± 0.29^***^	3.42 ± 0.48^***^	1.53
		4°C	1.85 ± 0.55^***^	3.02 ± 0.45^***^	1.63
		with EDTA	7.47 ± 1.31^*^	8.98 ± 0.90	1.20

* P < 0.05;

**P < 0.01;

*** *P < 0.001 (compared with control)*.

#### Effect of temperature

As depicted in Figure 2, the harmaline and harmine transport across the Caco-2 cell monolayers at the concentration of 2 μM was markedly reduced because *P*_*appAB*_ was changed from (27.72 ± 1.06) × 10^−6^ cm/s to (7.59 ± 0.33) × 10^−6^ cm/s for harmaline and from (18.78 ± 1.26) × 10^−6^ cm/s to (1.58 ± 0.57) × 10^−6^ cm/s for harmine when lowering the temperature from 37 to 4°C (*P* < 0.001). Simultaneously, the harmaline and harmine transport across the MDCK cell monolayers was also obviously reduced, the *P*_*appAB*_-values of harmaline and harmine at 37°C were significantly higher (3.77- and 2.49-folds) than those at 4°C (*P* < 0.001; Table 2), so did the opposite direction. The decreased *P*_*appAB*_ and *P*_*appBA*_ at 4°C might indicate that the transport was energy-dependent because decreasing the temperature would slow down cellular metabolism (Duan et al., 2014). However, further studies are necessary to confirm the energy dependence of the Caco-2 and MDCK cells for lowering the temperature may also slow down passive diffusion.

#### Effect of EDTA

In order to investigate the potential paracellular permeability of harmaline and harmine, EDTA (5 mM) was used to transiently open the functional barrier of TJ. The cellular TJ was modified by EDTA to remove Ca^2+^ ions from the medium via chelation. The TEER-values decreased, indicating the cell junctions had opened, and the TEER-values were below 100 Ω cm^2^ after EDTA treatment. Table 2 listed the *P*_*appAB*_-values of harmaline and harmine in both directions across the Caco-2 and MDCK monolayers with or without pretreatment with EDTA. Opening these junctions, the *P*_*appAB*_ and *P*_*appBA*_-values of harmaline and harmine across the Caco-2 cell monolayers were increased (*P* < 0.05; Table 2). After exposure of the MDCK cell monolayers to 5 mM EDTA at both sides, the harmaline and harmine transport was also increased because *P*_*appAB*_ was changed from (20.36 ± 1.82) × 10^−6^ to (29.43 ± 1.45) × 10^−6^ cm/s for harmaline and from (4.60 ± 0.25) × 10^−6^ to (7.47 ± 1.31) × 10^−6^ cm/s for harmine (*P* < 0.05), and the *P*_*appBA*_ was increased from (21.03 ± 2.11) × 10^−6^ to (26.34 ± 0.93) × 10^−6^ cm/s for harmaline (*P* < 0.05) and from (7.32 ± 0.19) × 10^−6^ to (8.98 ± 0.90) × 10^−6^ cm/s for harmine (Table 2). Interestingly, the significant increase in harmaline flux was observed when the bidirectional experiment was conducted in the presence of EDTA, while for harmine, which mainly increased in the AP to BL direction (Table 2). This is indicative of paracellular passive diffusion as the primary pathway for the permeability of the tested drugs (especially for harmaline) across the cell monolayers (Zhang et al., 2014). Besides, *P*_*appBA*_ of harmine remained almost constant irrespective in the presence or absence of EDTA, suggesting that harmine permeates the Caco-2 and MDCK cell monolayers via transcellular route in the AP to BL direction.

#### Effects of influx transporters

Interestingly, the majority of *P*_*appAB*_-values in the AP to BL direction showed a statistical difference compared to the control, but no significant difference was found in the BL to AP direction in the experiments with inhibitors or substrates. Thus, the results were focused on the effects of inhibitors or substrates on harmaline and harmine transport in the AP to BL direction in the two cell monolayers.

**Figure 3:**
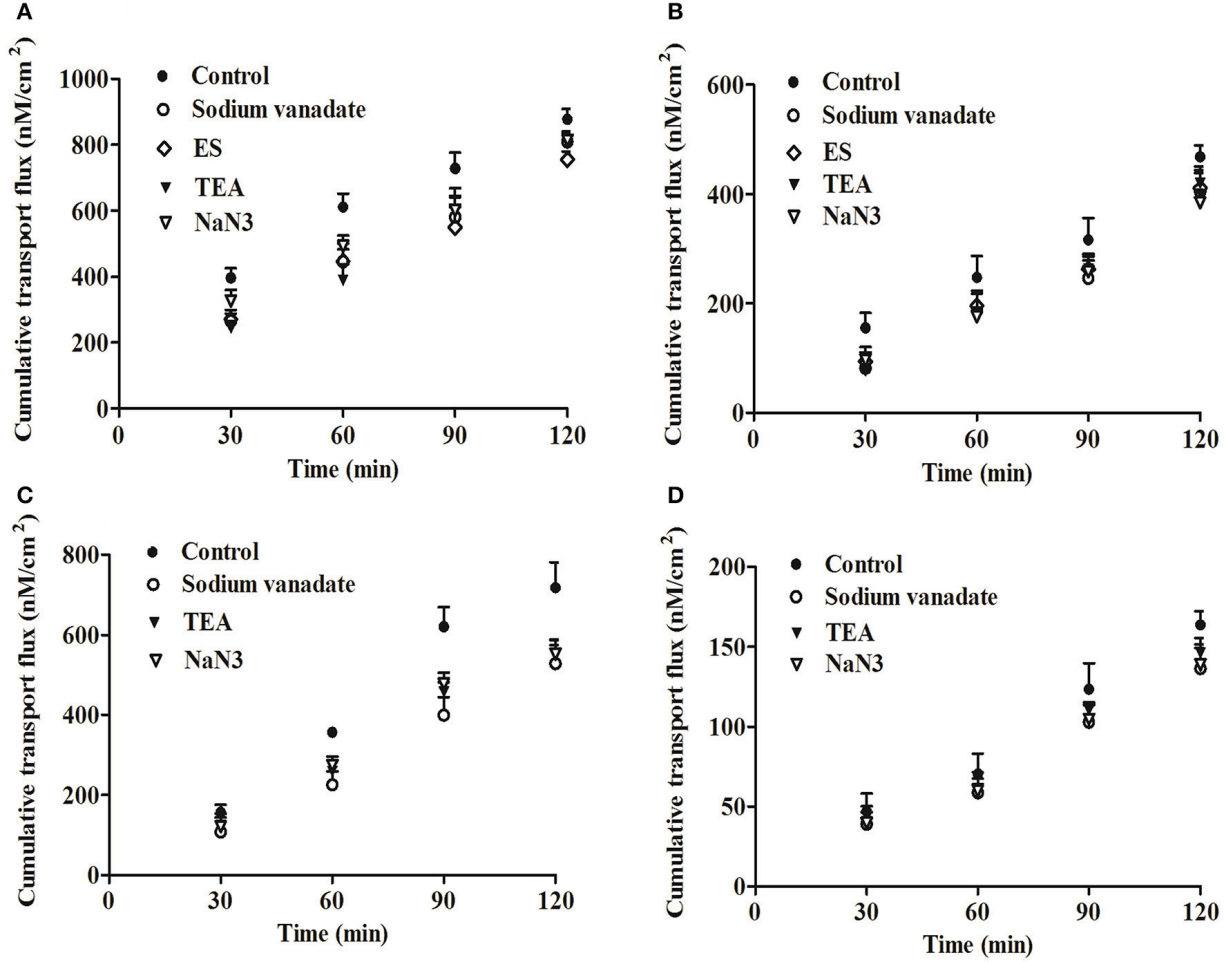
The cumulative transport fluxes of harmaline and harmine in the Caco-2 **(A,B)** and MDCK **(C,D)** cell monolayers in the AP to BL direction with various selective influx transporter inhibitors or substrates. Data represent the mean ± *SD* from three replicates.

Figure 3 and Tables 3, 4 presented the data from transport experiments using various selective influx transporter inhibitors or substrates. The investigations of harmaline and harmine influx mechanism utilized cimetidine and ES (OATs and OATPs inhibitors, respectively) on both sides. As Figure 3 and Table 3 indicated, no significantly decrease of harmaline and harmine transport amount at each sampling points, as well as *P*_*app*_ and *ER*, occurred after cimetidine addition (*P* > 0.05) during the experiment, implying that OATs did not cause the harmaline and harmine influx. However, the harmaline and harmine absorption was significantly decreased when 50 μM (Figure 3, *P* < 0.01) ES was added on the AP side; the *P*_*appAB*_ decreased from (27.48 ± 0.18) × 10^−6^ to (24.33 ± 0.49) × 10^−6^ cm/s for harmaline (*P* < 0.01) and from (18.52 ± 2.06) × 10^−6^ to (14.64 ± 1.31) × 10^−6^ cm/s for harmine (*P* < 0.05), respectively, while the *ER* increased from 0.95 to 1.12 for harmaline and from 1.59 to 1.94 for harmine (Table 3). Therefore, harmaline and harmine might be transported into the Caco-2 cells via OATPs. Moreover, TEA is the OCTs/OCTNs substrate behaves also as inhibitor, and it also inhibited the absorption of harmaline and harmine, and the permeability in the AP to BL direction was obviously decreased (Figure 3 and Table 3, *P* < 0.01), while *ER* was increased from 0.95 to 1.13 for harmaline and from 1.59 to 1.88 for harmine. To further investigate the ATP-mediated transport of harmaline and harmine, different substrates or inhibitors were added at both sides. NaN_3_ (10 mM, an ATP inhibitor) and sodium vanadate (50 μM, ATPase Na^+^/K^+^-dependent inhibitor) dramatically reduced the transport of harmaline and harmine, and the permeability in the AP to BL direction was obviously decreased (Figure 3 and Table 3, *P* < 0.01), while *ER* was increased. To investigate the involvement of MCTs, SGLT1 and PEPT1, the model inhibitors phloretin, phloridzin, and glycylsarcosine were used, respectively. However, 0.3 mM phloretin, 0.5 mM phloridzin and 10 mM glycylsarcosine, the concentration sufficient to inhibit MCTs, SGLT1, and PEPT1, had no effect on harmaline and harmine transport (Table 3). For the MDCK monolayers, the similar results were obtained except OATPs (Table 4), which might due to the quite low expression of OATPs in the MDCK cells (Volpe, 2011). In summary, harmaline and harmine might be the substrate of OATPs and OCTs/OCTNs influx transporters.

**Table 3:** Inhibitory effects on the bidirectional harmaline and harmine transport in the Caco-2 cell monolayers (mean ± *SD, n* = 3).

**Drug**	**Transporter**	**Inhibitors/Substrates**	**Concentration (mM)**	***P_*appAB*_* (1 × 10^−6^ cm/s)**	***P_*appBA*_* (1 × 10^−6^ cm/s)**	***ER***	**Modulatory effect**
Harmaline	Batch 1	Control	/	27.48 ± 0.18	26.19 ± 4.02	0.95	/
	MDR1	Verapamil	0.1	27.32 ± 1.33	25.68 ± 1.55	0.94	−
	MDR1	Quinidine	0.1	27.54 ± 1.35	26.44 ± 1.47	0.96	−
	BCRP	Ko143	0.01	28.03 ± 1.70	26.15 ± 0.48	0.93	−
	BCRP	Apigenin	0.025	27.85 ± 2.09	26.04 ± 1.43	0.94	−
	MRP2	MK571	0.05	27.90 ± 1.31	25.82 ± 0.71	0.93	−
	MRP2	Probenecid	0.2	27.22 ± 1.04	26.10 ± 1.02	0.96	−
	Na^+^	Sodium vanadate	0.05	23.12 ± 1.01^**^	26.89 ± 1.26	1.27	+
	OATs	Cimetidine	0.05	27.01 ± 1.47	26.16 ± 1.79	0.97	−
	OATPs	ES	0.05	24.33 ± 0.49^**^	26.41 ± 0.94	1.12	+
	OCTs/OCTNs	TEA	5	23.62 ± 1.01^**^	26.75 ± 1.58	1.13	+
	MCTs	Phloretin	0.3	27.77 ± 2.14	26.25 ± 0.91	0.95	−
	ATP	NaN_3_	10	22.75 ± 2.68^*^	26.18 ± 1.02	1.15	+
	SGLT1	Phloridzin	0.5	27.57 ± 2.40	26.07 ± 1.60	0.95	−
	PEPT1	Gly-Sar	10	27.73 ± 0.95	26.31 ± 1.18	0.95	−
Harmine	Batch 2	Control	/	18.52 ± 2.06	29.40 ± 1.38	1.59	/
	MDR1	Verapamil	0.1	18.43 ± 1.34	28.47 ± 3.77	1.54	−
	MDR1	Quinidine	0.1	18.69 ± 1.29	28.98 ± 3.96	1.55	−
	BCRP	Ko143	0.01	19.04 ± 3.67	27.96 ± 3.86	1.47	−
	BCRP	Apigenin	0.025	18.44 ± 4.36	28.40 ± 4.27	1.54	−
	MRP2	MK571	0.05	29.93 ± 2.14^**^	29.36 ± 2.73	0.98	+
	MRP2	Probenecid	0.2	23.45 ± 2.01^*^	27.93 ± 2.33	1.19	+
	Na^+^	Sodium vanadate	0.05	13.08 ± 0.93^*^	26.17 ± 1.78	2.00	+
	OATs	Cimetidine	0.05	18.65 ± 1.48	27.58 ± 1.54	1.48	−
	OATPs	ES	0.05	14.64 ± 1.31^*^	28.43 ± 1.89	1.94	+
	OCTs/OCTNs	TEA	5	14.96 ± 0.83^*^	28.05 ± 1.20	1.88	+
	MCTs	Phloretin	0.3	18.45 ± 1.42	27.37 ± 2.13	1.48	−
	ATP	NaN_3_	10	14.07 ± 0.39^*^	27.94 ± 1.76	1.99	+
	SGLT1	Phloridzin	0.5	19.11 ± 1.51	29.39 ± 1.30	1.54	−
	PEPT1	Gly-Sar	10	18.33 ± 1.28	27.86 ± 1.96	1.52	−

* P < 0.05;

** *P < 0.01 (compared with control)*.

*+, Denotes that the inhibitor or substrate has significant effect on the drug transport*.

*−, Denotes that the inhibitor or substrate has no significant effect on the drug transport*.

*/, Denotes no intervention*.

**Table 4:** Inhibitory effects on the bidirectional harmaline and harmine transport in the MDCK and MDCK-MRP2 cell monolayers (mean ± *SD, n* = 3).

**Cell**	**Drug**	**Transporter**	**Inhibitors/Substrates**	**Concentration (mM)**	***P_*appAB*_* (1 × 10^−6^ cm/s)**	***P_*appBA*_* (1 × 10^−6^ cm/s)**	***ER***	**Modulatory effect**
MDCK	Harmaline	Batch 1	Control	/	20.36 ± 1.82	21.03 ± 2.11	1.03	/
		MDR1	Verapamil	0.1	20.96 ± 1.30	21.95 ± 2.64	1.05	−
		MDR1	Quinidine	0.1	20.06 ± 0.76	22.46 ± 1.12	1.12	−
		BCRP	Ko143	0.01	20.08 ± 0.41	23.37 ± 0.73	1.16	−
		BCRP	Apigenin	0.025	20.44 ± 2.19	23.73 ± 1.11	1.16	−
		MRP2	MK571	0.05	19.66 ± 0.65	22.57 ± 0.14	1.15	−
		MRP2	Probenecid	0.2	21.01 ± 2.16	21.86 ± 1.21	1.04	−
		Na^+^	Sodium vanadate	0.05	16.16 ± 1.45^*^	20.37 ± 0.22	1.26	+
		OATs	Cimetidine	0.05	19.17 ± 1.29	19.21 ± 1.33	1.00	−
		OATPs	ES	0.05	19.45 ± 3.23	20.89 ± 0.79	1.07	−
		OCTs/OCTNs	TEA	5	16.48 ± 1.10^*^	22.75 ± 0.29	1.38	+
		MCTs	Phloretin	0.3	20.34 ± 0.71	23.31 ± 1.40	1.15	−
		ATP	NaN_3_	10	16.22 ± 0.84^*^	22.78 ± 1.65	1.40	+
		SGLT1	Phloridzin	0.5	15.96 ± 0.96^*^	20.59 ± 1.19	1.29	+
		PEPT1	Gly-Sar	10	20.88 ± 2.90	21.42 ± 1.96	1.03	−
	Harmine	Batch 2	Control	/	4.60 ± 0.25	7.32 ± 0.19	1.59	/
		MDR1	Verapamil	0.1	4.59 ± 1.34	7.67 ± 0.21	1.67	−
		MDR1	Quinidine	0.1	4.43 ± 0.32	7.39 ± 0.32	1.67	−
		BCRP	Ko143	0.01	4.50 ± 0.48	7.05 ± 0.16	1.57	−
		BCRP	Apigenin	0.025	4.48 ± 0.24	6.83 ± 1.01	1.53	−
		MRP2	MK571	0.05	5.14 ± 0.10^*^	6.67 ± 0.37	1.30	+
		MRP2	Probenecid	0.2	5.21 ± 0.26^*^	7.24 ± 0.27	1.39	+
		Na^+^	Sodium vanadate	0.05	3.87 ± 0.27^*^	5.22 ± 0.97^*^	1.35	+
		OATs	Cimetidine	0.05	4.74 ± 0.13	7.43 ± 0.62	1.57	−
		OATPs	ES	0.05	4.59 ± 0.22	7.17 ± 0.68	1.56	−
		OCTs/OCTNs	TEA	5	4.15 ± 0.10^*^	6.96 ± 0.24	1.68	+
		MCTs	Phloretin	0.3	4.51 ± 0.16	7.47 ± 0.27	1.66	−
		ATP	NaN_3_	10	4.02 ± 0.18^*^	5.94 ± 0.63^*^	1.48	+
		SGLT1	Phloridzin	0.5	3.89 ± 0.36^*^	6.27 ± 0.78	1.61	+
		PEPT1	Gly-Sar	10	4.59 ± 0.12	7.50 ± 0.34	1.63	−
MDCK-MRP2	Harmaline	Batch 1	Control	/	24.29 ± 2.72	32.38 ± 3.81	1.33	/
		MRP2	MK571	0.05	31.80 ± 1.37	40.90 ± 5.48	1.34	−
		MRP2	Probenecid	0.2	32.93 ± 2.23	41.93 ± 5.58	1.28	−
	Harmine	Batch 2	Control	/	3.55 ± 0.40	8.18 ± 0.98	2.31	/
		MRP2	MK571	0.05	20.80 ± 1.49^***^	11.80 ± 1.25	0.57	+
		MRP2	Probenecid	0.2	10.05 ± 1.69^**^	8.62 ± 1.05	0.86	+

* P < 0.05;

** P < 0.01;

*** *P < 0.001 (compared with control)*.

*+, Denotes that the inhibitor or substrate has significant effect on the drug transport*.

*−, Denotes that the inhibitor or substrate has no significant effect on the drug transport*.

*/, Denotes no intervention*.

#### Effects of efflux transporters

As illustrated in Tables 3, 4, the results from transport studies performed using various selective efflux transporter inhibitors or substrates. Verapamil and quinidine (100 μM, MDR1 inhibitors) were chosen because they are more selective for MDR1 than other efflux transporter inhibitors. The transport amount of harmaline and harmine at 120 min was not changed (Tables 3, 4, *P* > 0.05), implying that harmaline and harmine were not the MDR1 substrate under our experimental conditions. Similar experiments with verapamil and quinidine, the harmaline and harmine efflux was unaffected by BCRP inhibitors Ko143 (10 μM) and apigenin (25 μM). As displayed in Tables 3, 4, the transport amount of the tested drugs at 120 min and permeability were almost unchanged by Ko143 and apigenin treatment (*P* > 0.05). It suggested that both MDR1 and BCRP transporters were not involved in harmaline and harmine secretion.

**Figure 4:**
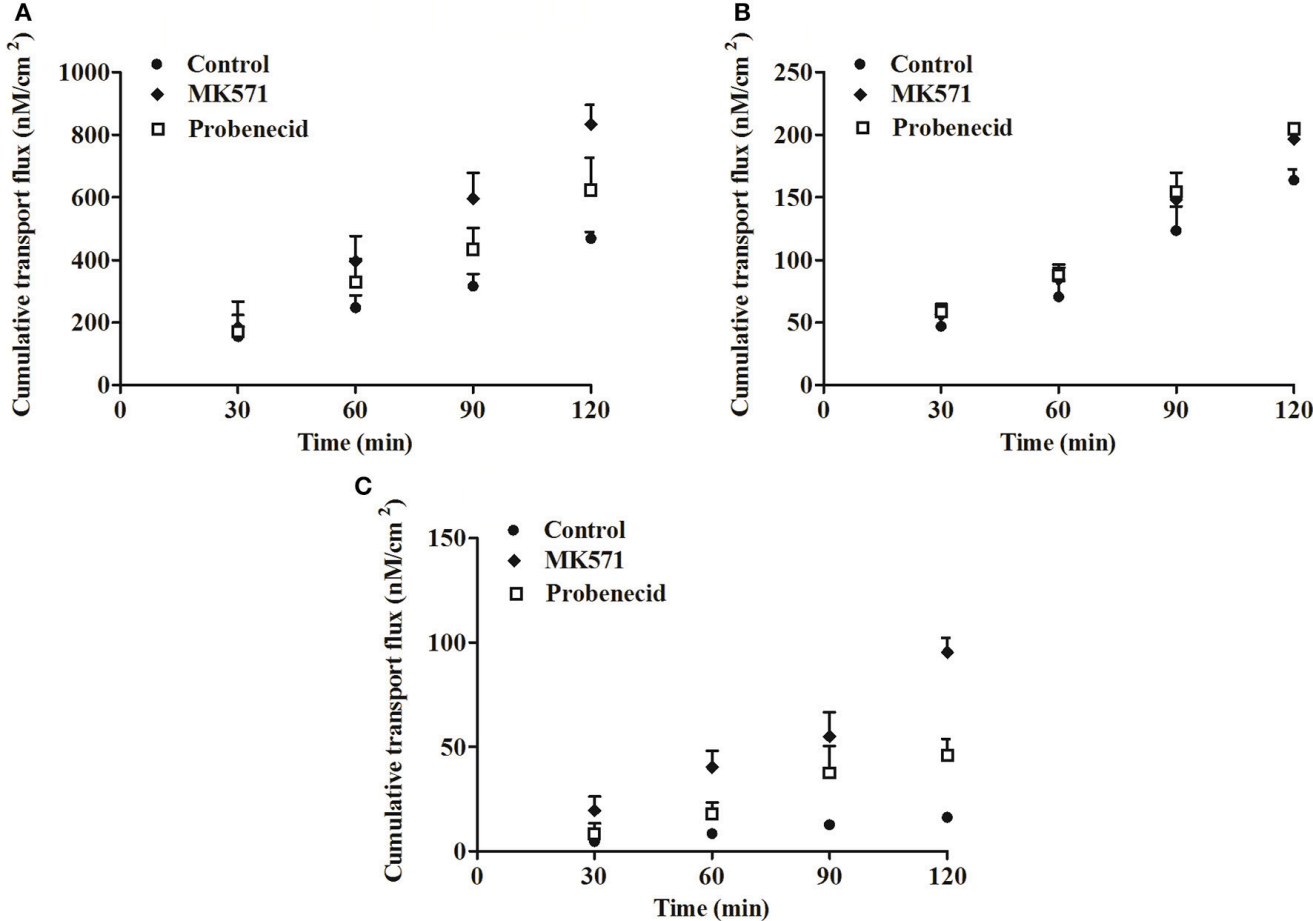
The cumulative transport fluxes of harmine in the Caco-2 **(A)**, MDCK **(B)**, and MDCK-MRP2 **(C)** cell monolayers in the AP to BL direction with various selective efflux transporter inhibitors or substrates. Data represent the mean ± *SD* from three replicates.

However, when MK571 (a typical MRP2 inhibitor) was added to the AP side in the Caco-2 cell monolayers, the *P*_*appAB*_ of harmine at 120 min was increased (1.62-folds), and the *ER*-value was decreased from 1.59 to 0.98 (Figure 4A and Table 3, *P* < 0.01), implying that MRP2 transporter governed the secretion of harmine. This result was validated by the values of *P*_*appAB*_ and *ER* when the efflux of harmine was inhibited by probenecid. Probenecid is an MRP2 inhibitor that markedly increased harmine transport from AP to BL direction with the *P*_*appAB*_-value increased (1.27-folds; Figure 4A and Table 3, *P* < 0.05). However, both inhibitors of MRP2 (MK571 and probenecid) had no effect on harmaline transport, and the *P*_*appAB*_ and *ER* in harmaline transport were similar to the control in the AP to BL direction (Tables 3, 4, *P* > 0.05). Therefore, the inhibitory effect of MK571 and probenecid implied that MRP2 was primarily responsible for the harmine efflux in the AP to BL direction.

In summary, among the three selected ATP-binding cassette (ABC) transporter inhibitors (MDR1, BCRP, MRP2), especially MRP2, revealed a high affinity for harmine during harmine secretion because MK571 and probenecid enhanced the permeability of harmine in the AP to BL direction. Additionally, the same results have been verified in other cells (MDCK and MDCK-MRP2; Table 4 and Figures 4B,C, *P* < 0.05).

### Vesicular transport

**Figure 5:**
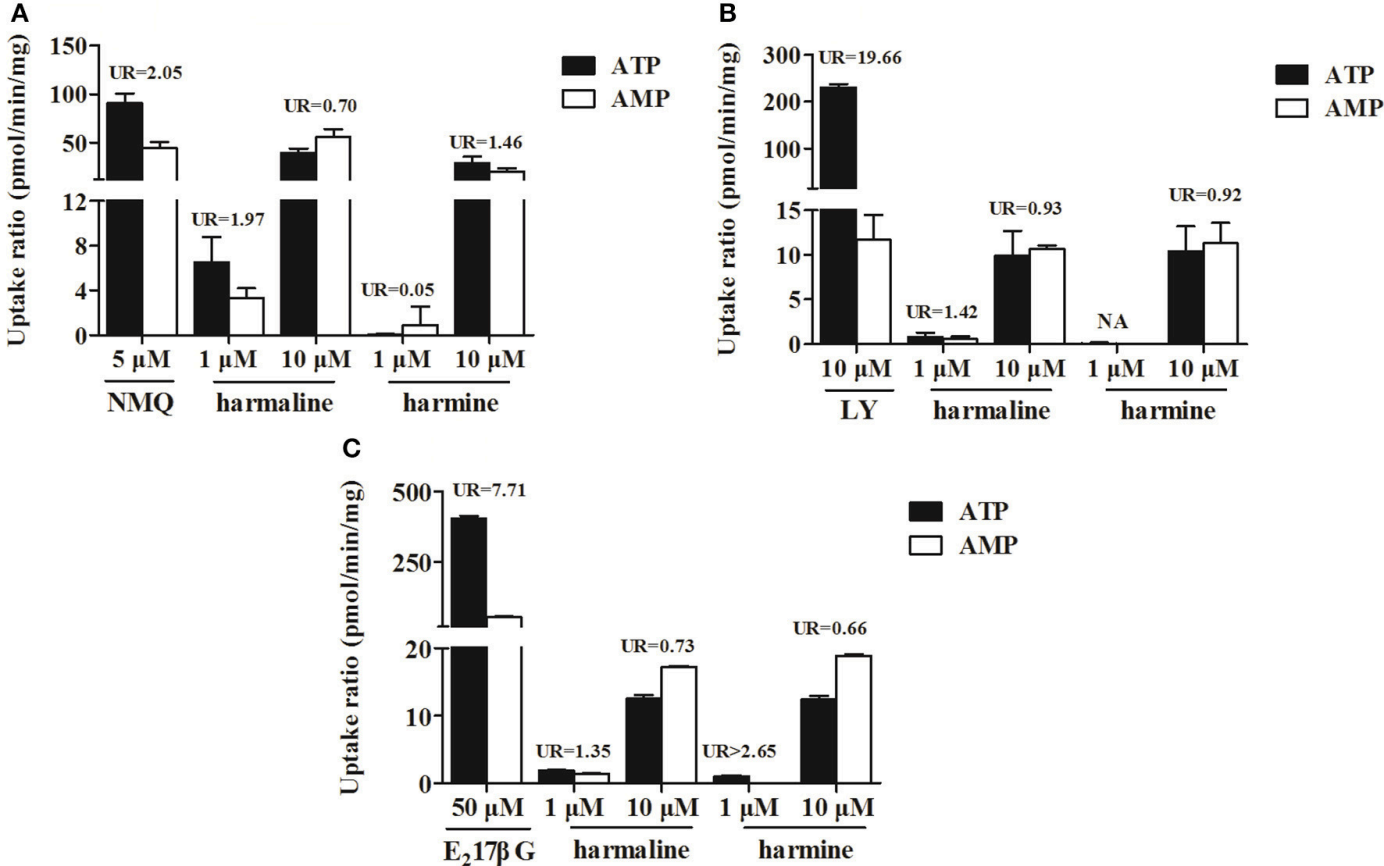
The uptake ratios (UR) of three probes (NMQ, LY, E_2_17βG), harmaline and harmine on membrane vesicles with over-expressed transporters MDR1 **(A)**, BCRP **(B)**, and MRP2 **(C)**. Data represent the mean ± *SD* from three replicates.

To validate the transport properties of the commercial Sf9 membrane vesicles expressing human MDR1, BCRP, and MRP2, the three probes *N*-methylquinidine (NMQ), lucifer yellow (LY) and estradiol-17 β-glucuronide (E_2_17βG) were used. As shown in Figure 5, the uptake ratios (UR) of the three probes were 2.05, 19.66, and 7.71 respectively, which indicated that the membrane vesicles could be used in further studies (all UR-values were above 2.00). It also showed that the UR-values of harmaline at 1 and 10 μM were <2.00 in all membrane vesicles expressing human MDR1, BCRP, and MRP2. Particularly, the UR-value of harmine at 1 μM was >2.65 in the membrane vesicles expressing human MRP2, which implying harmine might be the substrate of MRP2 (Figure 5C).

### Western blot analysis

**Figure 6:**
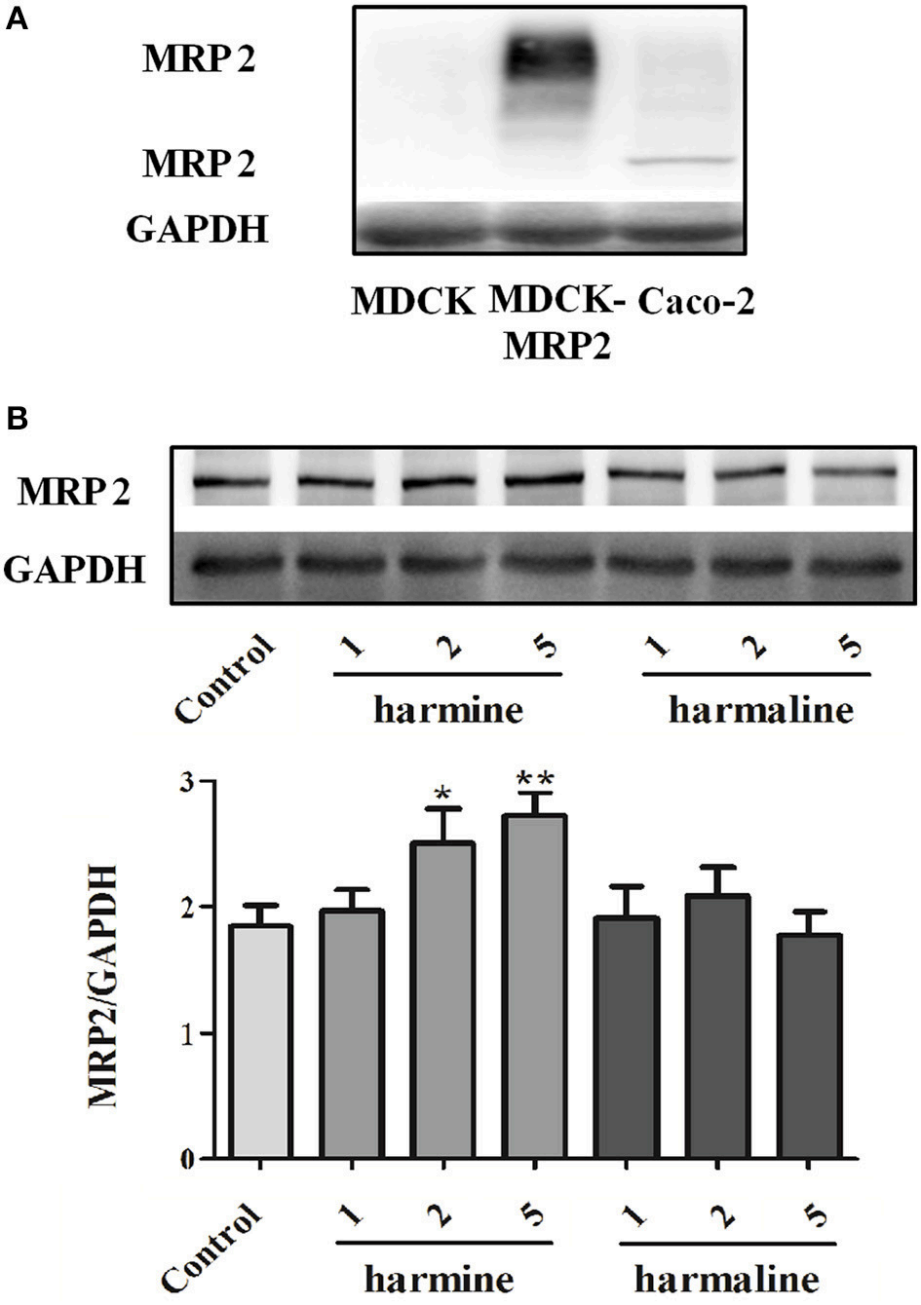
The protein expression of MRP2 in the Caco-2, MDCK, and MDCK-MRP2 cells **(A)** and the effects of harmaline and harmine **(B)**. Data represent the mean ± *SD* from three replicates ^*^P < 0.05; ^**^P < 0.01 (compared with control).

The protein expression of MRP2 in the Caco-2, MDCK, and MDCK-MRP2 cells were determined by western blot. As depicted in Figure 6A, MRP2 significantly expressed, low expressed and over-expressed in the Caco-2, MDCK, and MDCK-MRP2 cells, respectively. Furthermore, it can be seen from Figure 6B, harmine (2 and 5 μM) could slightly up-regulate the expression of MRP2 compared with the control group, indicating that harmine might be the substrate of MRP2 and could probably inhibit the absorption of those components mediated by MRP2. However, harmaline at the tested concentrations (1, 2, and 5 μM) had no effect on the expression of MRP2, demonstrating that MRP2 was not responsible for harmaline transport.

### Metabolic stability of harmaline and harmine in human liver microsomes

**Figure 7:**
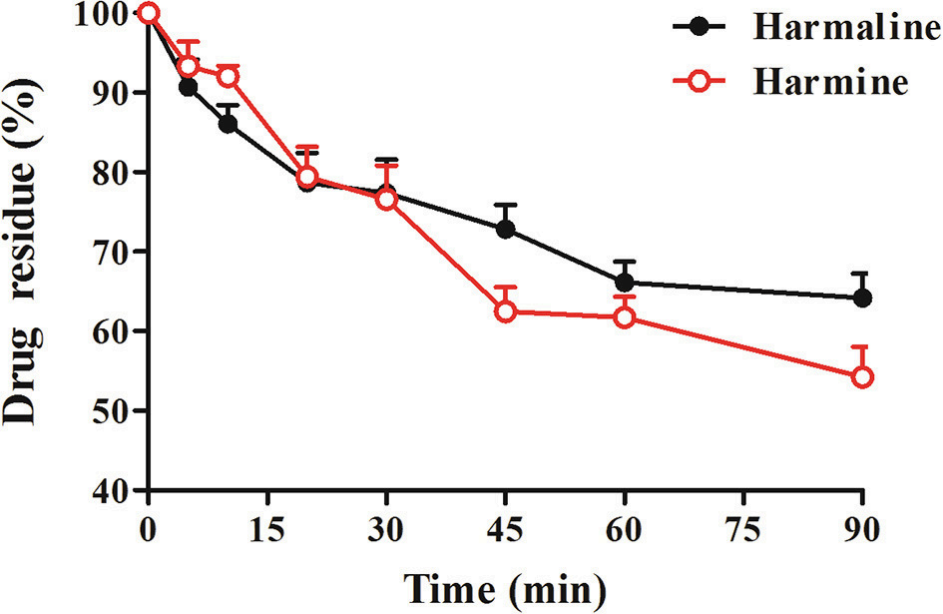
The residual percentage of harmaline and harmine in human liver microsomes. Data represent the mean ± *SD* from six replicates.

After incubating of harmaline or harmine at 2 μM, the residual percentage values of harmaline and harmine were 64.16 and 54.25%, respectively (Figure 7). The *T*_*1/2*_-value was 147.45 min for harmaline and 99.00 min for harmine in human liver microsomes. The *CL*_*int*_-value was 2.8 mL/min/mg for harmine, which was ~1.49-folds greater than that of harmaline (1.88 mL/min/mg), suggesting that harmaline is more stable in human liver microsomes than harmine.

### Pharmacokinetics

**Figure 8:**
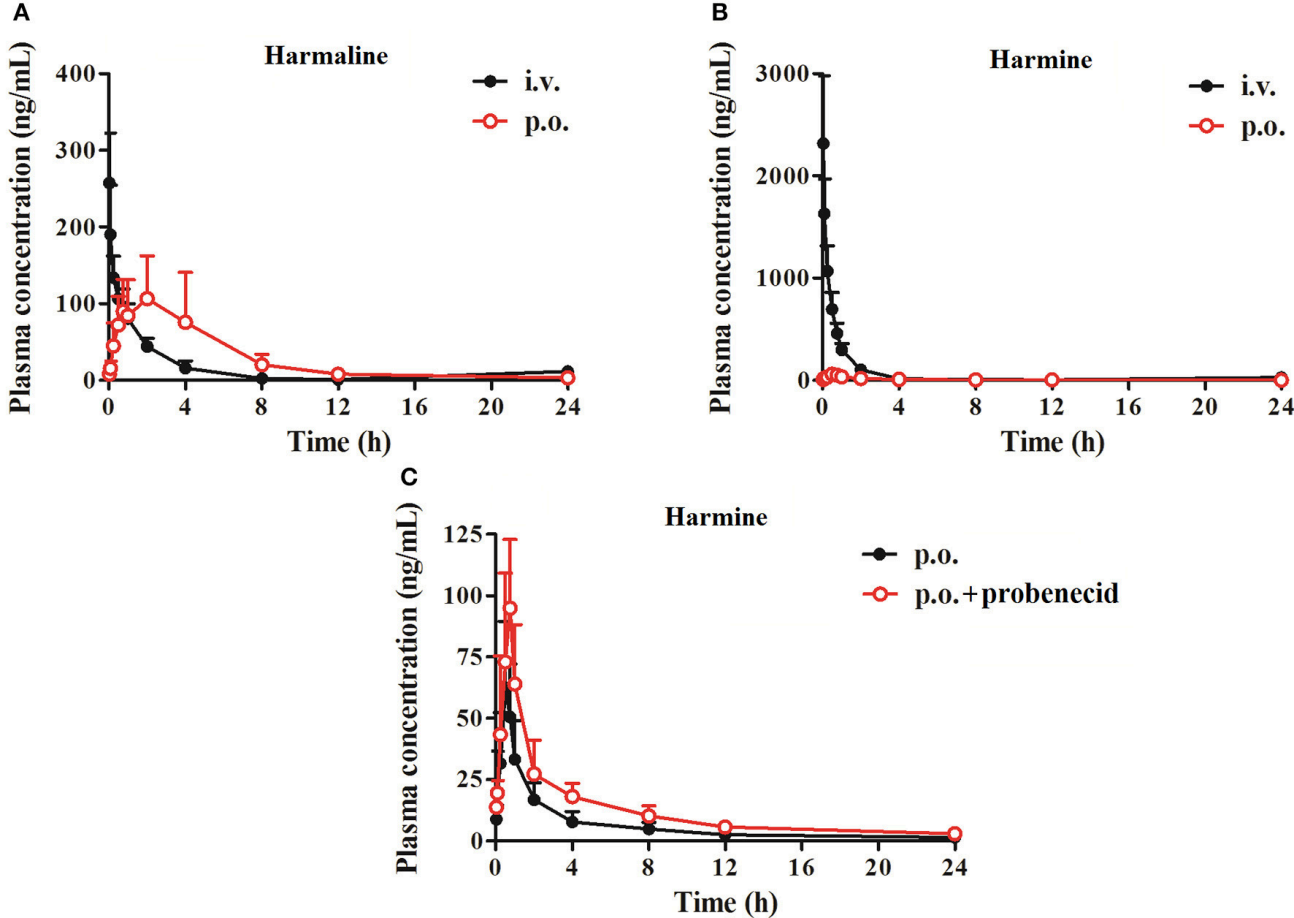
Mean plasma concentration-time curves of harmaline **(A)** and harmine **(B)** by intragastric (40.0 mg/kg) or intravenous (3.3 mg/kg) administration and harmine (40.0 mg/kg) co-administration with an MRP2 inhibitor probenecid (20.0 mg/kg) **(C)** by intragastric administration. Data represent the mean ± *SD* from eight replicates.

The pharmacokinetic parameters of harmaline and harmine were all obtained after oral and intravenous administration at the dose of 40.0 and 3.3 mg/kg, respectively. Figure 8 presents the mean plasma concentration-time curves of harmaline and harmine after administration in rats. Table 5 gives the corresponding pharmacokinetic parameters of harmaline and harmine following administration in rats.

**Table 5:** Pharmacokinetic parameters of harmaline and harmine in rats after intragastric and intravenous administration of harmaline, harmine, and harmine co-administration with an MRP2 inhibitor probenecid (mean ± *SD, n* = 8).

**Parameters**	**Harmaline (i.v.)**	**Harmaline (p.o.)**	**Harmine (i.v.)**	**Harmine (p.o.)**	**Harmine+Probenecid (p.o.)**
*C_*max*_* (ng/mL)	257.53 ± 64.88	117.80 ± 59.01	2319.54 ± 663.12	67.05 ± 34.29	98.91 ± 31.10^*^
*T_*max*_* (h)	0.03 ± 0.00	1.76 ± 1.11	0.03 ± 0.00	0.56 ± 0.13	0.79 ± 0.12^**^
*K_*e*_* (h^−1^)	0.23 ± 0.16	0.20 ± 0.17	0.30 ± 0.16	0.05 ± 0.02	0.05 ± 0.02
*K_*d*_* (h^−1^)	0.91 ± 0.64	0.39 ± 0.25	1.30 ± 0.98	0.36 ± 0.32	0.45 ± 0.31
*K_*a*_* (h^−1^)	1.26 ± 0.83	0.71 ± 0.38	2.71 ± 1.24	0.63 ± 0.42	0.73 ± 0.46
*T_*1/2e*_* (h)	4.05 ± 1.39	5.13 ± 1.52	3.06 ± 1.78	4.73 ± 0.71	8.61 ± 3.05^**^
*T_*1/2d*_* (h)	1.57 ± 1.25	2.40 ± 1.20	1.11 ± 1.06	3.77 ± 2.83	2.42 ± 1.89
*T_*1/2a*_* (h)	1.24 ± 1.02	1.74 ± 1.13	0.31 ± 0.15	1.72 ± 1.44	1.23 ± 0.65
*AUC_(0−*t*)_* (ng•h/mL)	298.52 ± 64.28	643.98 ± 327.52	1263.20 ± 237.60	154.17 ± 46.50	291.64 ± 70.40^***^
*AUC_(0−∞)_* (ng•h/mL)	306.77 ± 64.35	666.15 ± 321.79	1272.72 ± 239.33	183.56 ± 57.35	348.74 ± 76.67^***^
*MRT* (h)	2.53 ± 1.42	6.24 ± 3.18	1.01 ± 0.30	4.41 ± 1.03	13.70 ± 3.54^***^
*V_*d*_* (mL/kg)	94106.39 ± 102377.44	705974.45 ± 595531.26	10442.93 ± 5748.79	4761642.39 ± 1571772.10	2464535.13 ± 990154.63^**^
*CL* (mL/h/kg)	10364.66 ± 2636.13	74669.25 ± 41320.69	2448.11 ± 410.80	233439.53 ± 56204.60	118004.32 ± 24599.81^***^
*F*%	-	17.11 ± 10.09	-	1.09 ± 0.27	2.14 ± 0.59^***^

* P < 0.05;

** P < 0.01;

*** P < 0.001 (compared with harmine-p.o.)

Following administration by gastric *gavage*, harmaline, and harmine can be absorbed into blood circulation with a short *T*_*max*_ of 0.56 ± 0.13 h and low *C*_*max*_ of 67.05 ± 34.29 ng/mL for harmine, and a relative long *T*_*max*_ of 1.76 ± 1.11 h and high *C*_*max*_ of 117.80 ± 59.01 ng/mL for harmaline after a single oral dose of 40.0 mg/kg in rats (Figure 8 and Table 5). The plasma concentration vs. time curve of harmaline and harmine produced a slow phase of decrease with *T*_*1/2e*_ of 5.13 ± 1.52 h and 4.73 ± 0.71 h, respectively after dosing, which presented the same trend with intravenous dosage. To ascertain whether harmine is the substrate of MRP2, harmine was orally co-administration with probenecid (an MRP2 inhibitor). As presented in Figure 8 and Table 5, the *C*_*max*_, *T*_*max*_, *T*_*1/2e*_, *AUC*_(0−*t*)_, *AUC*_(0−∞)_, *MRT*-values of harmine in probenecid co-administration group were significantly higher than those of harmine administration group (Table 5, *P* < 0.05, *P* < 0.01, or *P* < 0.001). On the contrary, the *V*_*d*_- and *CL*-values were obviously lower than those of harmine administration group (Table 5, *P* < 0.01 or *P* < 0.001). It was worth noting that the *F*-value of harmine was increased 1.96-folds after probenecid co-administration, which further demonstrated that harmine might act as a substrate of MRP2.

### Excretion in rats

The cumulative excretion of harmaline and harmine in urine and feces after oral administration of 40.0 mg/kg was presented in Table 6. Harmaline and harmine in rat urine were detectable with low concentration within 72 h after oral dosing, while none of harmaline and harmine could be found in feces within 48–72 h. Harmaline and harmine were poorly excreted via urine and feces, and four metabolites for harmaline (harmine, harmalol, harmol, M279) and two metabolites for harmine (harmol, M279) were found in rats. The cumulative excretion was 5.05 ± 4.12% for harmaline and 0.69 ± 0.36% for harmine in urine and feces within 72 h after intragastric dosing. Considering the combination of intact drugs and their metabolites in urine and feces, about 16.04 ± 5.45% for harmaline and 28.18 ± 8.24% for harmine were recovered, which suggested that harmine was easily metabolized than harmaline in rats *in vivo* after oral administration. The low recovery rate of harmaline and harmine was probably due to they transformed into other undetected metabolites, which need to be further confirmed.

**Table 6:** Urinary and fecal cumulative excretion of harmaline and harmine in rats following oral administration of harmaline and harmine (40.0 mg/kg; mean ± *SD, n* = 8).

**Drugs**	**Analytes**	**Cumulative excretion in urine (%)**			**Cumulative excretion in feces (%)**			**SUM**
		**24 h**	**48 h**	**72 h**	**24 h**	**48 h**	**72 h**	
Harmaline	Harmaline	4.00 ± 4.36	4.71 ± 4.20	4.85 ± 4.17	0.14 ± 0.10	0.19 ± 0.12	0.19 ± 0.12	5.05 ± 4.12
	Harmalol	1.16 ± 0.81	2.03 ± 2.12	2.30 ± 2.36	1.73 ± 1.01	1.87 ± 1.04	1.89 ± 1.04	4.18 ± 2.67
	Harmine	0.03 ± 0.01	0.04 ± 0.01	0.05 ± 0.01	0.01 ± 0.01	0.02 ± 0.01	0.02 ± 0.01	0.07 ± 0.01
	Harmol	0.23 ± 0.42	0.50 ± 0.55	0.65 ± 0.56	1.24 ± 0.51	1.64 ± 0.73	1.70 ± 0.75	2.35 ± 0.91
	M279	2.53 ± 0.82	3.84 ± 0.83	4.29 ± 0.88	0.08 ± 0.05	0.10 ± 0.06	0.10 ± 0.06	4.39 ± 0.88
	SUM	7.94 ± 5.21	11.22 ± 5.48	12.13 ± 5.63	3.21 ± 1.54	3.81 ± 1.75	3.91 ± 1.76	16.04 ± 5.45
Harmine	Harmine	0.02 ± 0.02	0.02 ± 0.03	0.03 ± 0.03	0.57 ± 0.37	0.65 ± 0.36	0.65 ± 0.36	0.69 ± 0.36
	Harmol	2.51 ± 2.82	4.63 ± 4.53	5.42 ± 5.19	5.66 ± 2.96	7.69 ± 3.33	8.38 ± 3.47	13.80 ± 5.71
	M279	9.89 ± 3.81	11.58 ± 3.47	12.99 ± 4.18	0.55 ± 0.66	0.60 ± 0.68	0.71 ± 0.68	13.69 ± 4.33
	SUM	12.42 ± 5.10	16.24 ± 6.17	18.44 ± 7.76	6.78 ± 3.53	8.92 ± 3.70	9.74 ± 3.80	28.18 ± 8.24

## Discussion

Central nervous system (CNS) diseases are major health issues and are often associated with disability or death. In the past few decades, the two analogs harmaline and harmine have been reported to show potential for treating Alzheimer's disease, Parkinson's disease, depression and other CNS disorders (Li et al., 2017). A number of researchers have conducted some studies on the transport, metabolism, pharmacokinetics, pharmacological and toxicology properties of harmaline and harmine (Li et al., 2017). In 2004, it was revealed that β-carboline alkaloids harmaline, harmine, harmalol, harmol, and harmane demonstrated moderate to high efflux rates and permeability coefficients in the concentration range of 250–500 μM, and followed a concentration dependent passive diffusion mechanism (Khan et al., 2004). However, according to previous studies, these alkaloids especially harmaline and harmine have showed remarkable cytotoxicity and significantly inhibited tumor cell growth with apoptotic effect (Picada et al., 1997; Lamchouri et al., 2012; Li et al., 2017). Hence, it is necessary to give preference to their cytotoxicity investigation when conducting the transport study of these alkaloids.

In the current study, the results of cytotoxicity assay confirmed that none of harmaline (up to 25 μM) and harmine (up to 5 μM) showed toxicity or decreased cell viability in the Caco-2 and MDCK cells. When drug concentration was higher than 100 μM for harmaline and 25 μM for harmine, it would produce notable toxicity (Figure S3). Nevertheless, the concentrations of the alkaloids were ranged from 250 to 500 μM in the study conducted by Khan et al. (2004), which were far exceeded the safe dosage and would result in damage to the cells and change the activity of transporters or the permeability of the Caco-2 and MDCK cell monolayers and ultimately lead to an inconsistent result in the transport characteristics of tested drugs compared with the safe dosage. Consequently, the purpose of this study was to clarify the intrinsically transport characteristics and mechanisms of harmaline and harmine, two analogous β-carboline alkaloids, across the intestinal Caco-2 and canine renal MDCK cell monolayers under the non-toxic concentration range (1, 2, and 5 μM).

In the present study, harmaline and harmine exhibited concentration-dependent permeability profile in the Caco-2 and MDCK cell monolayers (Figure 1). The permeability of harmaline from the AP to BL was almost equal to that from the BL to AP, indicating a non-polarized transport of harmaline. However, the permeability of harmine from the AP to BL was obviously lower than that from the BL to AP, indicating an active transport of harmine. The permeability of harmaline and harmine was slightly lower than that of propranolol, a transcellular flux marker with *P*_*appAB*_-value of (41.90 ± 3.30) × 10^−6^ cm/s, suggesting that harmaline and harmine were effectively absorbed by a transcellular pathway through the Caco-2 and MDCK cells (Shen et al., 2015). The transport of harmaline and harmine was notably decreased when experiments were performed at 4°C or in the presence of NaN_3_ and sodium vanadate (ATP inhibitor and ATPase Na^+^/K^+^-dependent inhibitor, respectively; Figure 2, 3 and Tables 2–4). It was indicated that harmaline and harmine were the energy-Na^+^-dependent system when they were absorbed. Besides, the permeability in the absorptive direction was significantly reduced at lower pH, implicating a pH-dependent absorption of harmaline and harmine. Additionally, this might be partially due to their physicochemical properties. Harmaline and harmine are weakly basic compounds with the pKa of 4.4 and 7.7 (calculated by ACD/I-Lab) respectively, and the *P*_*app*_-values of harmaline were higher than those of harmine at the same pH, since it possesses a lower pKa than harmine. Furthermore, they will be mostly in the form of ionized species in weakly acidic medium (pH 5.5) and consequently the passive transcellular route plays a minor role. It is well-known that un-ionized form compound is easier to transmembrane transport than ionized form. Therefore, the *P*_*app*_-values of harmaline and harmine were lower in pH 5.5 than those in pH 7.4 in the Caco-2 and MDCK cells. Accordingly, studies have demonstrated that the basic drugs (such as metoprolol, timolol) had much higher permeability at pH 7.4 than 5.0 (Balimane et al., 2006).

To further investigate the probable role in transporters-mediated absorption of harmaline and harmine, the effects of various compounds on the uptake in the Caco-2 and MDCK cells were examined. Results verified that OATs, MCTs, SGLT1, and PEPT1 had no effect on the transport of harmaline and harmine, while the inhibitor or substrate of OATPs and OCTs/OCTNs markedly reduced the cumulative transport fluxes of harmaline and harmine in the Caco-2 cells (Figure 3 and Table 3). For the influx transporters of harmaline and harmine in the MDCK cells, which were consistent with those of the Caco-2 cells except for OATPs (Figure 3 and Table 4). It was pointed out by Volpe (2011) that the MDCK cells mainly expressed canine OCTs, PEPT1, and MCTs uptake transporters, while scarcely expression of OATPs and consequently ES (an inhibitor of OATPs) had no effect on the absorption of harmaline and harmine in the MDCK cell monolayers (Table 4). Generally, OCTs/OCTNs are specific for cationic compounds, while OATPs are specific for anionic ones. However, our results indicated that harmaline and harmine were absorbed might be mediated by both OATPs and OCTs/OCTNs. Notably, as reviewed by van Montfoort et al. (2003), OATPs have a broad substrate specificity mediating transport of various compounds, including some organic cations (for instance, an alkaloid *N*-methyl-quinine, rocuronium, etc,). The structures of β-carboline alkaloids resemble *N*-methyl-4-phenyl-1,2,3,6-tetrahydropyridine (MPTP), and studies have showed that OCT1 and OCT2 are vital for MPTP transfer across the blood-brain barrier (Lin et al., 2010). Comprehensive analysis above, the influx protein carriers OATPs and OCTs/OCTNs may be involved in the transport of harmaline and harmine, and which needs to be further validated in other cell lines with over-expressed OATPs and OCTs/OCTNs transporters.

Membrane transporters, particularly the ABC efflux transporters MDR1, BCRP, and MRP2, have been known to affect the extent of absorption and oral bioavailability of numerous drugs. From the current studies, MDR1 and BCRP had no effect on the efflux of harmaline and harmine in the Caco-2 and MDCK cells. Nevertheless, the efflux of harmine was effectively inhibited by MK571 or probenecid, inhibitors of MRP2 (Shen et al., 2015), suggesting the involvement of MRP2 in the efflux of harmine (Figure 4 and Tables 3, 4). Moreover, the results were validated using the MDCK cell with over-expressed MRP2 (MDCK-MRP2; Figure 4C and Table 4). Apart from these results obtained in cell-based assays, inverted membrane vesicles with over-expressed transporters provide a high throughput and convenience way to study the transport mechanism of drugs. It can be seen from Figure 5, the UR-value of harmine at 1 μM was higher than 2.00 (UR > 2.65) in the membrane vesicles expressing human MRP2, suggesting MRP2 was responsible for harmine transport (Figure 5C). According to the results of western blot analysis, harmine (2 and 5 μM) could slightly up-regulate the expression of MRP2, which demonstrated that harmine was a substrate of MRP2 (Figure 6B).

Additionally, *in vitro* metabolic stability and *in vivo* excretion investigations clearly manifested that harmine was more inclined to metabolize into other metabolites than harmaline (Figure 7 and Table 6), which may be one of the reason inducing the significant difference in bioavailability of the two analogs. Furthermore, the pharmacokinetic study confirmed that the high exposure of harmine was found with the increased absorption and reduced elimination after co-administration with probenecid, which further verified that harmine might act as a substrate of MRP2 (Figure 8 and Table 5).

**Figure 9:**
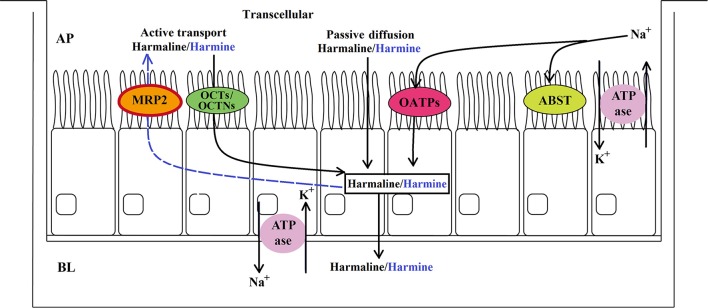
Proposed transport pathways of harmaline (black) and harmine (black and blue) in the Caco-2 and MDCK cell monolayers.

In summary, harmaline and harmine might be absorbed from the AP to BL side by two mechanisms: (a) mainly transcellular passive diffusion and (b) pH- and Na^+^-dependent transport which probably mediated by influx protein carriers belonging to the SLC family, in particular OATPs and OCTs/OCTNs. Harmine might be secreted from the BL to AP side by the ABC efflux transporter MRP2. The transport pathways of harmaline and harmine through the Caco-2 and MDCK cells under our experimental conditions are tentatively summarized in Figure 9. Particularly, harmine was more unstable and easily metabolized than harmaline. These findings would suggest that harmine not only appears to be an MRP2 substrate, but also possesses weak metabolic stability, and eventually leads to a low exposure and oral bioavailability. Totally speaking, these results could provide enough useful information to elucidate harmaline and harmine pharmacokinetics and drug-drug interactions, and more *in vivo* evaluations must be undertaken further.

## Conclusions

In conclusion, as two analogs, harmaline, and harmine are transported by a complicated process: (1) mainly transcellular passive diffusion was involved; (2) pH- and Na^+^-dependent transport mediated by SLC influx and ABC efflux transporters. An ATP-dependent influx mechanism was critical for harmaline and harmine transport process. Influx transporters, particularly OATPs and OCTs/OCTNs, were probably involved in harmaline and harmine transport. Efflux transporters, especially MRP2 was vital for harmine transport in the intestines. *In vitro* metabolic stability and *in vivo* excretion studies clearly verified that harmine was more unstable and easily metabolized than harmaline. All findings indicated that harmine appears to be a substrate of the efflux transporter MRP2 with weak metabolic stability, which ultimately results in an exposure difference compared with its analog harmaline. Further experiments will be necessary to verify if other influx transporters (such as MATEs), or the specific isoforms of OCTs/OCTNs and OATPs are involved in the transport of harmaline and harmine.

## Author contributions

Participated in research design: SL, XC, YM, YX, and CW; Conducted experiments: SL, YW, YZ, GD, and SQ; Performed data analysis: SL and CW; Wrote or contributed to the writing of the manuscript: SL and CW.

### Conflict of interest statement

The authors declare that the research was conducted in the absence of any commercial or financial relationships that could be construed as a potential conflict of interest.
