# Supplementary material for: Exposure Characteristics of the Analogous β-Carboline Alkaloids Harmaline and Harmine Based on the Efflux Transporter of Multidrug Resistance Protein 2
Source: Front Pharmacol. 2017 Aug 21;8:541. doi: 10.3389/fphar.2017.00541 (PMC5566973; doi:10.3389/fphar.2017.00541)
Supplement: Supplementary file 1 [file DataSheet1.DOC]

**Supplemental material**

**Exposure Characteristics of the Analogous β-Carboline Alkaloids Harmaline and Harmine Based on the Efflux Transporter of Multidrug Resistance Protein 2**

Shuping Li ^a^, Yunpeng Zhang ^a^, Gang Deng ^a^, Yuwen Wang ^a^, Shenglan Qi ^a^, Xuemei Cheng ^a,b^, Yueming Ma ^c^, Yan Xie ^d^, Changhong Wang ^a,b*^

^a^ *Institute of Chinese Materia Medica, Shanghai University of Traditional Chinese Medicine; The MOE Key Laboratory for Standardization of Chinese Medicines and The SATCM Key Laboratory for New Resources and Quality Evaluation of Chinese Medicine, 1200 Cailun Rood, Shanghai 201210, China*

^b^ *Shanghai R&D Centre for Standardization of Chinese Medicines, 199 Guoshoujing Road, Shanghai 201210, China*

^c^ *Laboratory of Pharmacokinetics, Shanghai University of Traditional Chinese Medicine, Shanghai 201203, China*

^d^ *Research Center for Health and Nutrition, Shanghai University of Traditional Chinese Medicine, Shanghai 201203, China*

*Correspondence to: Professors Chang-hong Wang, The Institute of Traditional Chinese Medicine, Shanghai University of Traditional Chinese Medicine, Shanghai 201203, China.

Tel: +86 21 51322511, Fax: +86 21 51322519, E-mail: [wchcxm@hotmail.com](mailto:wchcxm@hotmail.com) (C.H. Wang).

**Abstract**

Harmaline and harmine occur naturally in plants and are distributed endogenously in human and animal tissues. The two β-carboline alkaloids possess potential for treating Alzheimer’s disease, Parkinson’s disease, depression and other central nervous system diseases. However, studies have showed that the two compounds have similar structures but with quite different bioavailability. The aim of this study was to elucidate the exposure difference and characterize the *in vitro* transport, metabolism, and pharmacokinetic properties of harmaline and harmine. The results showed that the harmaline and harmine transport across the Caco-2 and MDCK cell monolayers was varied as the time, concentration, pH and temperature changed. The absorption of harmaline and harmine was significantly decreased when ES (OATPs inhibitor), TEA (OCTs/OCTNs substrate), NaN_3_ (adenosine triphosphate inhibitor) or sodium vanadate (ATPase Na^+^/K^+^-dependent inhibitor) was added. However, when given MK571 and probenecid (the typical MRP2 inhibitor), the *P_appAB_* of harmine was increased (1.62-folds and 1.27-folds), and the efflux ratio was decreased from 1.59 to 0.98 and from 1.59 to 1.19, respectively. In addition, the uptake ratio of harmine at 1 μM was greater than 2.65 in the membrane vesicles expressing human MRP2. Furthermore, harmine could slightly up-regulate the expression of MRP2, which implying harmine might be the substrate of MRP2. Particularly, the *CL_int_* value for harmine was approximately 1.49-folds greater than that of harmaline in human liver microsomes. It was worth noting that the *F* value of harmine was increased 1.96-folds after harmine co-administration with probenecid. To summarize, comprehensive analysis indicated that harmaline and harmine were absorbed by transcellular passive diffusion and a pH- and Na^+^-dependent mechanism might be mediated by OATPs and OCTs/OCTNs. MRP2 but MDR1 or BCRP might be involved in the transport of harmine. Furthermore, harmine was more unstable and easily metabolized than harmaline. All these findings suggested that harmine not only appears be an MRP2 substrate, but also possesses weak metabolic stability, and eventually leads to a low oral bioavailability. Taken together, the elucidated absorption, transport, metabolism as well as pharmacokinetic characteristics of harmaline and harmine provide useful information for designing delivery systems, pharmacological applications and avoiding drug-drug interactions.

***Keywords:*** β-Carboline alkaloid; Transport; Metabolism; Pharmacokinetic; MRP2

**
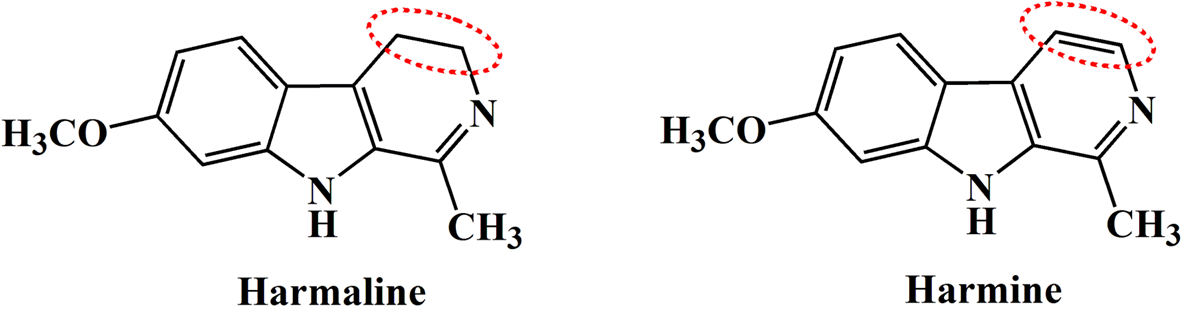
**

**Figure S1** The chemical structures of harmaline and harmine.

**
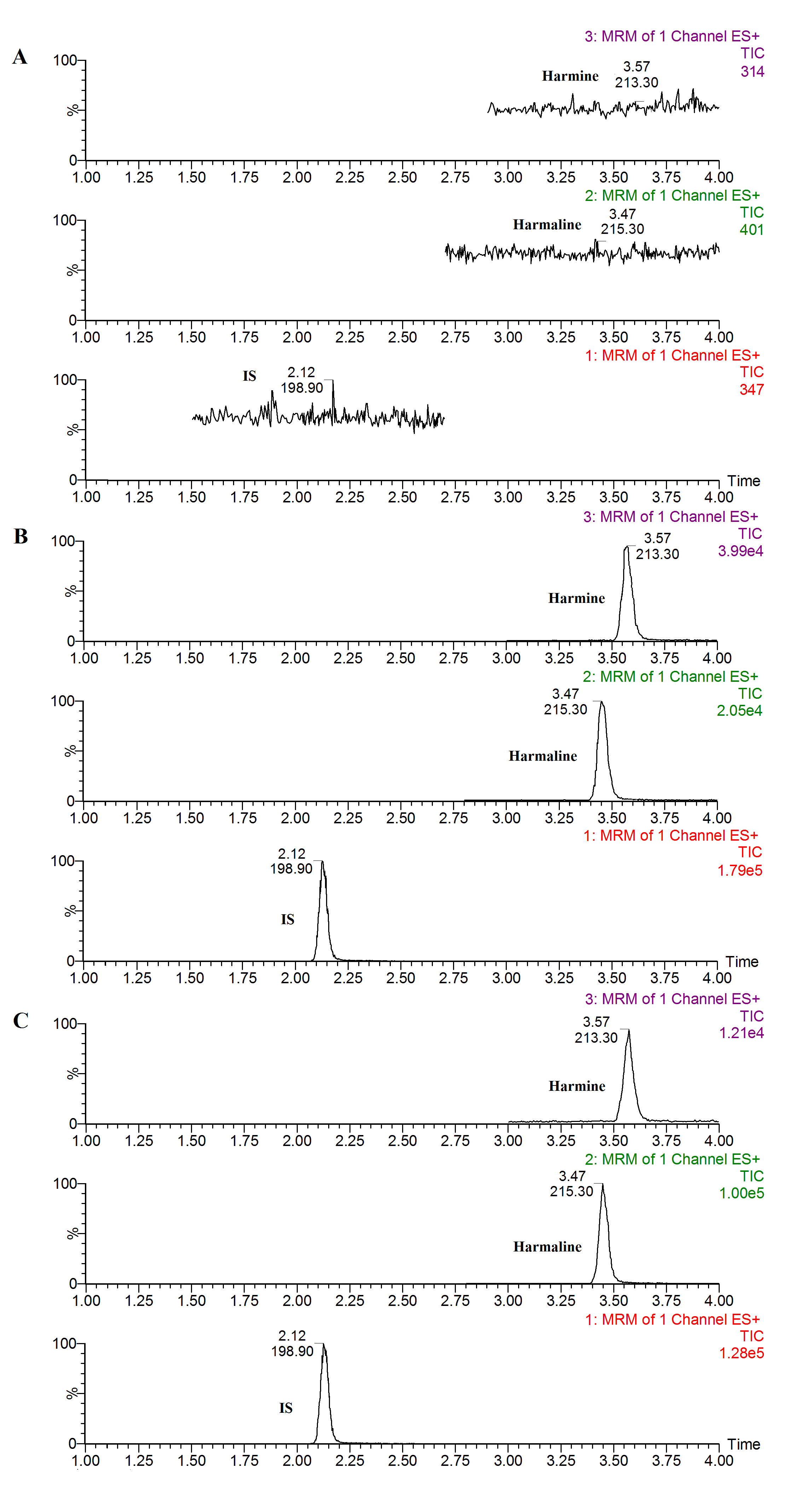
**

**Figure S2** The representative MRM chromatograms of blank HBSS (A); blank HBSS spiked with harmine, harmaline and IS (B); and HBSS sample spiked with IS after administration of harmine and harmaline (C).

**Figure S3** The cytotoxicity of harmaline and harmine in the Caco-2 (A) and MDCK (B) cells. Data represent the mean ± SD from three replicates.
